# Mechanics reveals the role of peristome geometry in prey capture in carnivorous pitcher plants (*Nepenthes*)

**DOI:** 10.1073/pnas.2306268120

**Published:** 2023-09-07

**Authors:** Derek E. Moulton, Hadrien Oliveri, Alain Goriely, Chris J. Thorogood

**Affiliations:** ^a^Mathematical Institute, University of Oxford, Oxford OX2 6GG, United Kingdom; ^b^University of Oxford Botanic Garden, Oxford OX1 4AZ, United Kingdom

**Keywords:** plant mechanics, biomechanics, mathematical model, carnivorous plants, *Nepenthes*

## Abstract

Carnivorous pitcher plants (*Nepenthes*) are a striking example of a natural pitfall trap. The trap’s slippery rim, or peristome, plays a critical role in insect capture via an aquaplaning mechanism that is well documented. While the peristome has received significant research attention, the conspicuous variation in peristome geometry across the genus remains unexplored. We examined the mechanics of prey capture using *Nepenthes* pitcher plants with divergent peristome geometries. Inspired by living material, we developed a mathematical model that links the peristomes’ three-dimensional geometries to the physics of prey capture under the laws of Newtonian mechanics. Linking form and function enables us to test hypotheses related to the function of features such as shape and ornamentation, orientation in a gravitational field, and the presence of “teeth,” while analysis of the energetic costs and gains of a given geometry provides a means of inferring potential evolutionary pathways. In a separate modeling approach, we show how prey size may correlate with peristome dimensions for optimal capture. Our modeling framework provides a physical platform to understand how divergence in peristome morphology may have evolved in the genus *Nepenthes* in response to shifts in prey diversity, availability, and size.

## Significance

Pitcher plants (*Nepenthes*) produce an astonishing array of leaf-derived traps into which prey (typically insects) slide from a rim (the peristome). How prey capture varies across this genus is a mystery. We hypothesized that the ability to capture an insect is connected to the peristome geometry and relative size. We demonstrated this connection by examining the physics of prey capture under the laws of Newtonian mechanics. Our analysis suggests that a diversity of peristomes in *Nepenthes* evolved in response to variation in prey capture.

Carnivorous plants evolved various forms of leaf-derived traps that attract, capture, retain, kill, and digest animal prey, as a mode of survival in nutrient-poor environments. *Nepenthes* is a tropical genus of carnivorous pitcher plants that produce specialized pitfall traps. Insects are attracted by lures such as coloration and nectar and become trapped when they “aquaplane” off the slippery pitcher rim (peristome), a surface structured with specialized ridges (1, 2), leading them to fall into a vessel of digestive fluid (3). The insects release nitrogen which gives the plants a strong selective advantage in environments where light and water are plentiful but nutrients are limiting (4).

The specialized trapping surfaces of carnivorous *Nepenthes* pitcher plants are receiving growing interest from biologists and engineers because of their strong biomimetic potential (5). For example, the slippery trapping surface of the *Nepenthes* pitcher has inspired slippery liquid-infused porous surfaces (SLIPS) which have exceptional wettability performance (6, 7). Yet despite research focused on the peristome as a key feature in the evolution of the trap, and as a source of inspiration to technologists, little is known about the mechanics of prey capture in *Nepenthes*, or how this varies among species.

To date, there are 183 accepted species of *Nepenthes* (POWO, 2023) and they show an astonishing diversity in pitcher morphology. Little is known about the prey trapped by most species in nature. Among the few species in the genus examined, diversity seems to mirror a range of nutrient acquisition strategies linked to habitat characteristics (8). For example, ants are a common form of prey in lowland habitats (9), whereas flying insects are often trapped by plants growing in mountain environments (10). More specifically, research in the last two decades has revealed that divergent pitcher morphology is linked to nutrient acquisition sources ranging from termites (9) and leaf litter (11) to mammalian feces (12, 13). Most recently, a species was reported from Borneo that produces pitchers underground (14). This diversity in pitcher function appears to be the result of an adaptive radiation driven by dietary shifts, analogous to well-known examples in animals, such as the diverse beak shapes of Darwin’s finches and the various adaptations of cichlid fish in the African Great Lakes (3). However, only a fraction of the diversity of *Nepenthes* has been examined, and we know little or nothing of the prey spectrum for most species.

**Fig. 1.**
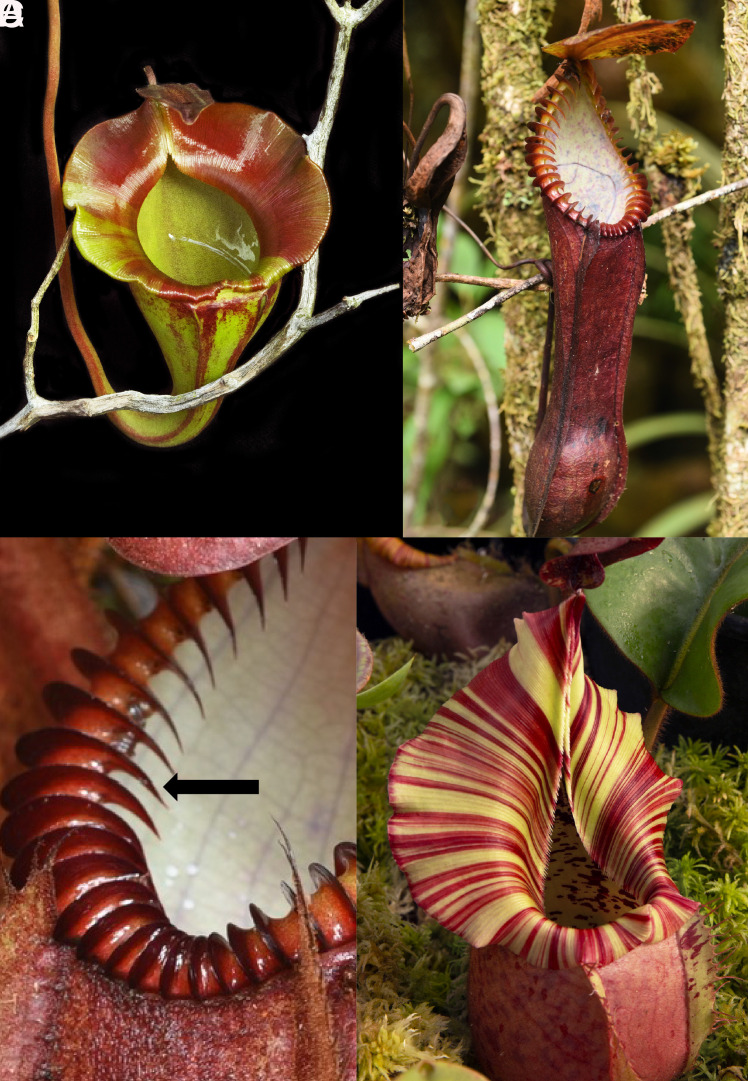
Divergent morphology in the genus *Nepenthes* shown by (*A*), the flat peristome of *N. jacquelineae*; (*B* and *C*) the prominent teeth (arrow) of *N. hamata*, and (*D*) the conspicuously flared peristome of *N. veitchii*. Photos (*A*) and (*D*) by Domonick Gravine; photos (*B* and *C*) by Jeremiah Harris.

**Fig. 2.**
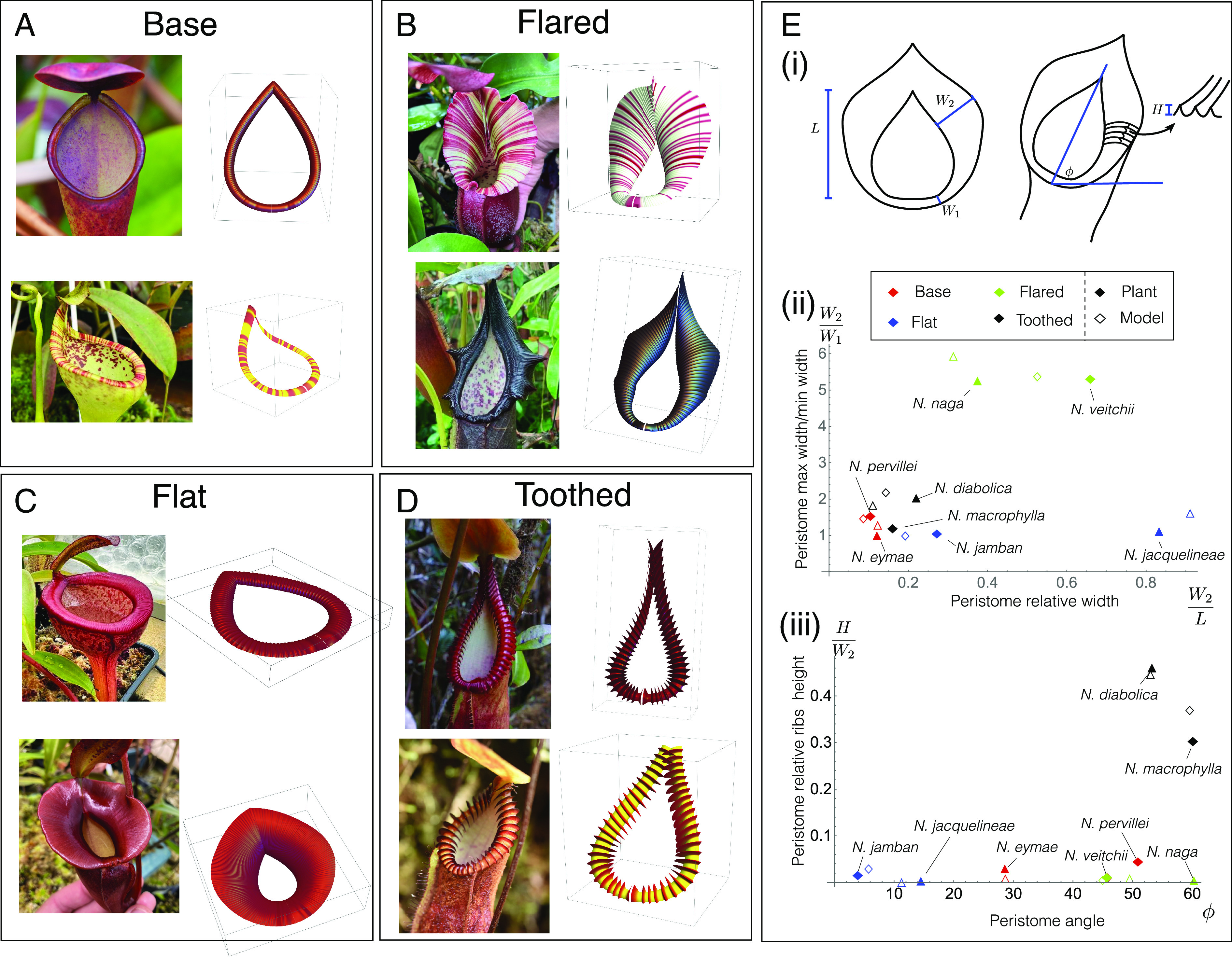
Variation in peristome geometry and mathematical reconstructions. We categorize peristomes into 4 categories: (*A*) Base geometry, exemplified by *N. pervillei* (*Top*) and *N. eymae* (*Bottom*); (*B*) Flared geometry, exemplified by *N. veitchii* (*Top*) and *N. naga* (*Bottom*); (*C*) Flat geometry, exemplified by *N. jamban* (*Top*) and *N. jacquelineae* (*Bottom*); and (*D*) Toothed geometry, exemplified by *N. macrophylla* (*Top*) and *N. diabolica* (*Bottom*). Mathematical surface reconstructions for each peristome are shown at *Right* for each species. Details on the mathematical construction process are given in *SI Appendix*, section 1. Quantification of surface properties is demonstrated in (*E*). From the 5 surface measures shown in (*E*), *i*, we define 4 (dimensionless) surface properties: peristome flaring is plotted against relative width in (*E*), *ii*; relative ribbing/teeth height is plotted against orientation angle in (*E*), *iii* for all sample species and model reproductions. *N. pervillei* photo by Ulrike Bauer; *N. eymae* photo by Sarracenia Northwest; *N. jamban*, *N. naga*, and *N. macrophylla* photos by Tom Bennet (https://www.tomscarnivores.com); *N. jacquelineae*, *N. veitchii*, and *N. diabolica* photos by Jeremiah Harris.

The general mechanism by which insects slide off the *Nepenthes* is well documented. A film of water stabilizes on the superhydrophilic surface (1). The surface is covered by a regular, hierarchical microstructure of parallel ridges, or channels (2, 5). These ridges guide prey into the trap in a controlled way through (5). Macroscopic ridges restrict lateral but enhance radial spreading of water, hence creating slippery chutes. Meanwhile, microscopic ridges ensure the watery film between the insects’ feet and the peristome remains stable, causing insects to aquaplane (2). These principles seem to be consistent across multiple species, indicating a common mechanism underlying insect aquaplaning. However, the gross morphology of peristomes is conspicuously diverse in size and geometry, ranging from cylindrical rims to highly ornate, fluted, and toothed structures (Fig. 1). This diversity can be linked to ecological niche. For example, *N. veitchii* [Fig. 2*B*] has an unusual life history: The plant clings to trees with the pitchers oriented such that the ventral surface is parallel to the tree surface. Meanwhile, species such as *N. macrophylla* and *N. diabolica* [Fig. 2*D*] produce pitchers, often half-buried in moss, with conspicuously toothed peristomes. Unfortunately, the prey spectrum of these species—like the majority of species—and the function of these structures, are poorly documented. Why peristomes are so variable and how their geometry relates to prey capture remain unknown.

Here, we present a mathematical framework to link divergent three-dimensional peristome geometries to the physics of prey capture. Linking form and function, we test the hypothesis that shape and ornamentation, orientation in a gravity field, presence of teeth, and peristome size, influence the diversity of prey capture in *Nepenthes*.

## 1. Mathematical Approach

Our objective is to develop a mathematical framework linking peristome geometry to prey capture to investigate whether the observed diversity in peristome geometry can be understood in simple physical terms relating to prey-capture functionality. Of the 183 known species, there exists a wide variety in peristome size and morphology. Here, we focus on three key geometric features of the peristome: i) the peristome width and presence and degree of peristome flaring—broad and often fluted, ii) the orientation, or tilt, of the peristome with respect to gravity, and iii) the presence of surface features such as ribbing or in extreme cases, teeth—prominent spine-like, parallel features. Based on these features, we classify *Nepenthes* peristomes into four categories that could be easily compared, as illustrated in Fig. 2*A*–*D*, Base, Flared, Flat, and Toothed:

- Base peristomes are thin with a roughly 45${}^{{}^{\circ}}$ tilt with respect to the vertical, and inconspicuous ribbing. A paradigm for this type is *N. pervillei*, a species from the Seychelles, established to be sister to all other species of *Nepenthes* (15). It is reasonable to assume that other, more ornate patterns of geometry, evolved from this ancestral state.
- Flared peristomes are similar to the base geometry distally (at the front) but flare out to varying degrees proximally (near the point of attachment to the lid).
- Flat peristomes have a similar geometry to Base, but with a wider rim. These peristomes are distinct from the flared ones in that they are more uniform in width. They are also characterized by a flatter orientation with respect to gravity compared with the other types which are tilted such that the proximal region is lower than the distal portion.
- Toothed peristomes also have a similar geometry to Base–thin and without flaring–but possess prominent ribs, so large that they are often referred to as “teeth,” protruding from the peristome and projecting into the pitcher interior. Despite their conspicuousness, their function is unknown.

The surface characteristics particular to each category may be quantified in terms of five basic measures denoted schematically in Fig. 2*E*, *i*: i) the interior peristome length (L); ii) the minimum ($W_{1}$) and maximum ($W_{2}$) peristome widths; iii) the angle of the peristome with respect to gravity (ϕ); and iv) the average ribbing height (H). From these, we define the peristome relative width $W_{2}/L$, the degree of flaring $W_{2}/W_{1}$, the prominence of ribs/teeth $H/W_{1}$, and the orientation ϕ. In Fig. 2*E*, *ii* and *iii*, we plot these values for each of the sample species (see *SI Appendix*, section 1C for details on parameter extraction), from which the distinctive features of each group are quantitatively apparent. (Peristome curvature is also relevant in prey capture but is not included as a measure here as it is less practical to define a single meaningful value that can be extracted from an image.)

To explore fully the potential functions of the features described above, we must first establish a robust mathematical framework that can describe accurately the diverse geometries involved. In *SI Appendix*, section 1, we have outlined a systematic procedure for creating parameterized mathematical surfaces that model various peristomes. The construction process consists in defining first a space curve giving the basic peristome shape; we then construct explicit cross-sectional shapes at discrete points along the peristome, through which we have fine control over local curvature and features such as flaring; interpolation between these cross-sections leads to construction of the full surface. The peristome shape and curvature profile were varied until a reasonable visual match with the chosen specimen was reached. This approach allows us to generate realistic peristome geometries that can be modified easily and continuously as needed as shown in Fig. 2. The characteristic measures defined above may also easily be extracted from the mathematical surfaces; these data points are included in Fig. 2 *E*, *ii* and *iii*, showing good proximity with the peristomes being represented. By employing this construction process, we can create a wide range of peristome shapes and configurations and investigate their properties and functions. For a given peristome type, we have a vector of parameters S that defines the peristome surface $\Sigma \subset R^{3}$.

Given a peristome surface Σ(S), we characterize prey capture capabilities by first considering the sliding of a point mass on the surface Σ as a function of surface wetness. Neglecting the deformation of the peristome due to the small mass of the insect, we assume that the peristome remains fixed and rigid. The first question is: Is an insect’s position p∈Σ on the surface stable under the force of gravity? This is a simple geometry problem that involves determining the local peristome orientation in the gravitational field using the normal vector n to Σ, and the coefficient of static friction μ.

The effect of increasing wetness is to reduce the stability of most positions. Therefore, our second question is crucial: If a position on the peristome is unstable, will the insect slide into or out of the pitcher? The dynamics of a point mass on the peristome is given by a system of differential equations that can be integrated in time until either the inner or outer edge of the peristome is reached. Points whose trajectory leads to the inside rim of the peristome will be deemed caught by the pitcher, contributing nutrients to the plant, while points whose trajectory leads to the outside rim will fall off the edge, contributing nothing.

Details outlining this procedure and our computational approach can be found in *SI Appendix*, section 2. Since we have an explicit surface parameterization, we can easily calculate surface stability and sliding dynamics and divide the surface Σ, for a given friction coefficient, into different nonintersecting regions of total area $A=A_{\text{stable}}+A_{\text{unstable}}=A_{\text{stable}}+A_{\text{in}}+A_{\text{out}}$ and:

$$\begin{array}{cc} & \Sigma_{\text{stable}}:\text{stable region of area}\;A_{\text{stable}};\\ & \Sigma_{\text{in}}:\text{unstable region, prey falls in, with area}\;A_{\text{in}};\\ & \Sigma_{\text{out}}:\text{unstable region, prey falls out, with area}\;A_{\text{out}}. \end{array}$$

Next, we use the above approach to analyze flaring, orientation, and ribbing features. It is important to highlight the modeling trade-off: The analysis in these sections is carried out on detailed and realistic geometries, but using a highly idealized and simplified description of the insect itself as a point mass. To complement this analysis, we present, in Section 5, a second model that takes into account the size of the prey.

## 2. The Benefits of a Flared Peristome

The pitcher plant species *N. veitchii* [Fig. 2*B*] has a striking peristome, which is broad and oblique. This peristome type is also observed in other species such as *N. nebularum*, *N. hurrelliana*, *N. naga*, and *N. robcantleyi*. However, the prey spectra of these species in their natural habitats remain undocumented, and the evolutionary drivers behind this peristome morphology are still unknown.

To gain insight into the potential benefits of a Flared peristome for prey capture, we first analyze the stability properties of the peristome surface as wetness increases. By examining the peristome geometry and its response to different levels of wetness, we can develop a better understanding of how this structure functions and how it may have evolved to suit the needs of the plant.

**Fig. 3.**
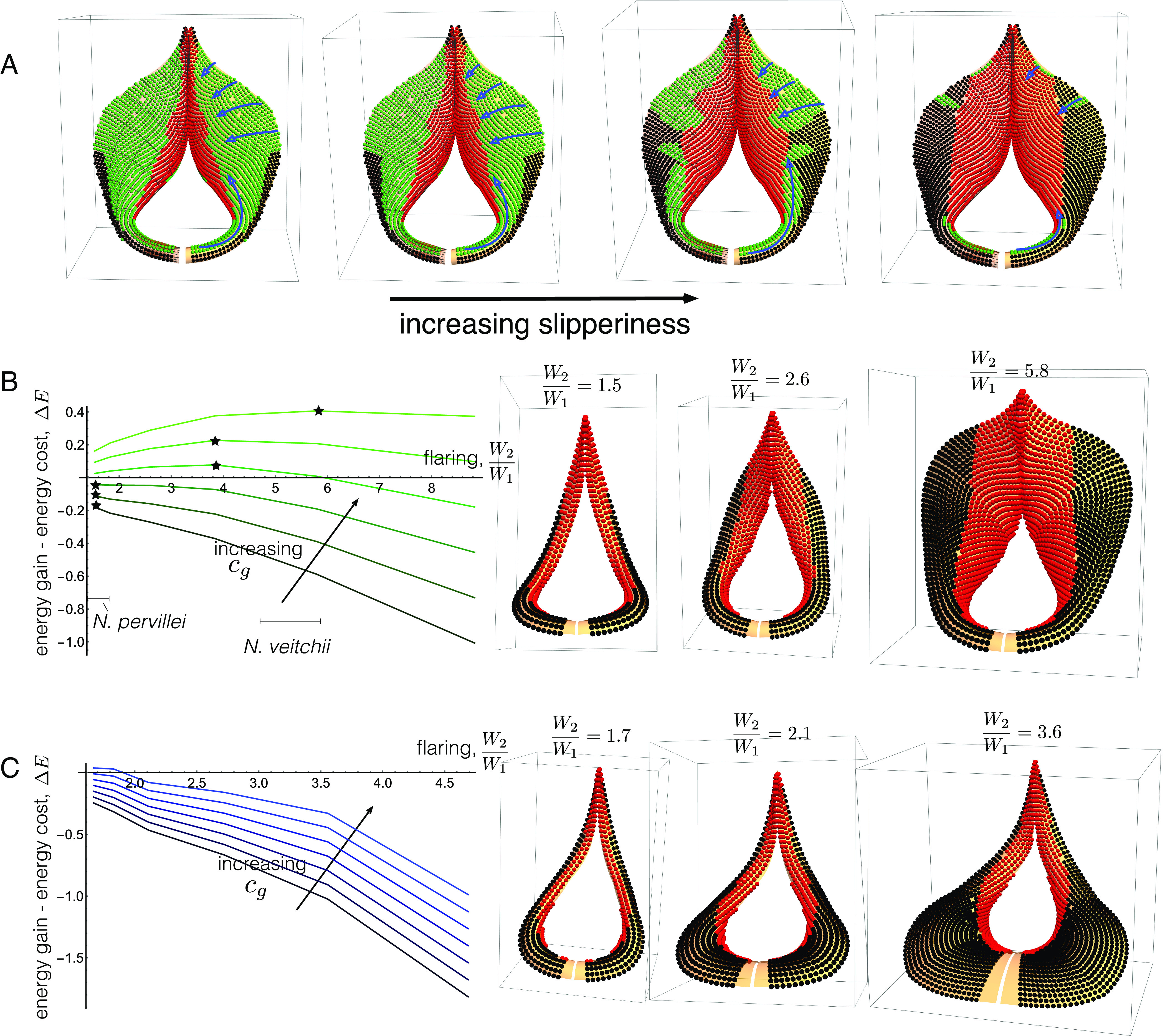
The impact of flaring on prey-capture. (*A*) Stability and capture properties of a Flared peristome as friction coefficient μ is decreased. Green points are stable, red points slide into the pitcher, and black points slide out. Stability “corridors”—stable paths from the edge of the peristome to the unstable inner rim—are highlighted with blue lines and arrows. (*B*) and (*C*): Net energy gain ΔE plotted for peristome flaring, $W_{2}/W_{1}$, and for different values of energy benefit parameter $c_{g}$, for normal flaring (*B*) and lower rim flaring (*C*). The point of maximum ΔE is denoted with a star. In (*B*), measured flaring values (given as an approximate range) for *N. veitchii* and *N. pervillei* are indicated. *Right*: The peristome geometry at indicated values of α, with fall-in and fall-out points shown in red and black, respectively.

In Fig. 3*A*, we present the result for our model of a Flared peristome, with each point of the surface colored according to the vantage of the insect giving both its stability and dynamic properties: Points in the region $\Sigma_{\text{stable}}$ are green (safe); points in $\Sigma_{\text{in}}$ are labeled red (unsafe), and points in $\Sigma_{\text{out}}$ are labeled black. The different surfaces correspond to differing degrees of “slipperiness”: The friction coefficient, denoted μ, decreases following the arrow, corresponding to a more slippery surface.

Naturally, as the surface becomes more slippery, a larger area becomes unstable; indeed $\Sigma_{\text{stable}}$ shrinks to a set of zero area in the limit of zero friction. It is also unsurprising that points on the inner rim, where the surface becomes nearly vertical, are red (the dynamics end with the prey falling in), with this red region $\Sigma_{\text{in}}$ expanding with increasing slipperiness. The black region, $\Sigma_{\text{out}}$, is “useless” to the plant, as prey located at these points will fall out of the pitcher. It is interesting to note that $\Sigma_{\text{out}}$ remains relatively small until very high slipperiness, and always has a smaller area than $\Sigma_{\text{in}}$.

Nectar glands are located near the inner rim of the peristome. Therefore, it is in this general direction that prey are likely to be attracted. Further, a recent study (16) presents a capture mechanism in which scout ants are able to walk on the peristome surface without sliding and falling in; these scout ants recruit workers, enabling a batch catch and thus greater benefit than if the scout ants had fallen in. In the context of these two points, Flared geometry may be adaptive for capturing walking prey such as ants. At low slipperiness, there are few black regions; thus the surface geometry provides a safe platform for scout ants to locate nectar, and subsequent worker ants to follow pheromone trails to the red region. As slipperiness increases, stable green “corridors” enable insects to walk from the outer edge of the peristome to the red region, as highlighted by blue arrows in Fig. 3*A*. Owing to the climbing habit of *N. veitchii*, the proximal portion of the peristome often touches the vertical axis of the supporting tree. Here, the flared peristome may act as a corridor to the pitfall trap—a form of shuttle for insects crawling up and down the tree.

### Energy Considerations.

A fundamental trade-off exists in carnivorous plants: Leaves are modified into traps at the expense of photosynthetic efficiency because the traits of an effective insect trap are incompatible with those of an efficient light trap (4). Our analysis of Flared peristomes indicates a similar trade-off between prey capture and cost associated with the production of a peristome. The peristome contributes little to photosynthesis and is costly to construct (17), suggesting a strong selective advantage to such a structure in a nutrient-stressed environment. Quantifying such trade-offs between peristome investment and prey capture with empirical data is challenging, not least since the identity of prey in nature is unknown for most species. Nevertheless, we gain insight into this problem by using a modeling approach in which we assume that the energetic benefit, denoted $E_{\text{gain}}$, is an increasing function of the capture surface area; that is,

[1] $$\begin{array}{c} E_{\text{gain}}=g\left(A_{\text{in}}\right), \end{array}$$

where g is a monotonically increasing function. This models the assumption that the benefit increases with the number of prey caught and that the number of prey caught increases with the area of peristome from which prey fall. The situation may be different for flying versus walking prey, given that flying prey can land anywhere on the surface while walking prey can only access stable edge portions as a starting point. Though given how little is known of the prey spectra of most species, we leave more detailed modeling for future work. Since $A_{\text{in}}$ depends on the friction coefficient μ, we compute $A_{\text{in}}$ in the case of a perfectly wetted surface (μ=0), for simplicity. We model the energetic cost as an increasing function of the total peristome area, that is,

[2] $$\begin{array}{c} E_{\text{cost}}=f\left(A\right)=f\left(A_{\text{in}}+A_{\text{out}}\right), \end{array}$$

the latter equality reflecting the fact that the stable area shrinks to zero when μ→0.

We can then define the net energy

[3] $$\begin{array}{c} \Delta E:=E_{\text{gain}}-E_{\text{cost}}=g\left(A_{\text{in}}\right)-f\left(A\right). \end{array}$$

We want to express ΔE as a function of a given peristome feature that may be varied through natural developmental mechanisms. Then, through evolution by natural selection, the feature may be expected to converge to the point where ΔE is maximal, or at least near to it (as other factors may impact the total evolutionary fitness). If changing a given feature decreases ΔE, we do not expect to see such changes in nature. Of course, it will depend on the specific form of the functions f and g. Here, we consider a generic form $g\left(x\right)=c_{g}x^{\beta_{g}}$, $f\left(x\right)=c_{c}x^{\beta_{c}}$, where the constants $c_{g}$ and $c_{c}$ characterize the energetic gain and cost, respectively, i.e., the impact of increased capture area and total area, while the exponents $\beta_{g}$ and $\beta_{c}$ characterize possible nonlinearity in the pathway between areas and energy.

We now examine flaring under this framework. Our construction method enables us to continuously vary the degree of flaring, from thin (as in Base) to a widely flared peristome, or even beyond what is observed in nature. Therefore, we express ΔE as a continuous function of the flaring parameter $W_{2}/W_{1}$ where $W_{2}/W_{1}$ ranges from 1.0 (unflared, similar to *N. pervillei*) to 9.0 (more flared than what we have measured on *N. veitchii*)—for details on continuously varying flaring, see *SI Appendix*, section 1. For a given $W_{2}/W_{1}$, we seed the peristome with a uniform distribution of point masses, integrate forward the dynamic trajectories, and compute the capture (and miss) areas as fractions of total area based on the number of trajectories leading to the inner (and outer) rim (details in *SI Appendix*, section 2). In Fig. 3*B*, we plot ΔE over a range of values of $W_{2}/W_{1}$ for varying choices of $c_{g}$, where we have fixed without loss of generality $c_{c}=1$, and with other parameters taken for simplicity to be $\beta_{c}=\beta_{g}=1$ (see also *SI Appendix*, section 2*D* for an analysis of how varying these parameters impacts the net energy). For each choice of $W_{2}/W_{1}$, the maximum of ΔE is denoted with a star. For low values of $c_{g}$, ΔE decreases monotonically with $W_{2}/W_{1}$. Here, the benefit from increased prey capture is relatively low: the cost of increased total area outweighs the gain from increased capture area; for a species with these parameters, it would not be energetically favorable to increase flaring. For an increased $c_{g}$, however, ΔE exhibits nonmonotonic behavior, and indeed with an interior maximum, the degree of flaring to which our model would predict selection pressures will drive the feature.

One great advantage of modeling is that it allows us to investigate features that are not found in nature. For instance, in Fig. 3*C*, we repeated the same analysis, but with flaring along the bottom rim of the peristome. Such peristome geometries are not observed in nature, and our energy model demonstrates why this might be the case: The increased area at the bottom rim does not contribute to prey capture, as prey located there will fall out of the pitcher when slippery. Thus, increasing flaring in this manner does not result in a net benefit. This is evidenced by the fact that ΔE decreases as flaring increases for all the tested values of $c_{g}$, rendering it a nonadaptive feature.

## 3. Peristome Orientation

Next, we consider the orientation of the peristome with respect to the vertical. Peristome orientation varies conspicuously across the genus from near-horizontal, for example, in *N. jamban* and *N. jacquelineae* [Fig. 2*C*], to an orientation of ca. 45${}^{{}^{\circ}}$, for example *N. veitchii* and *N. naga* [Fig. 2*B*].

**Fig. 4.**
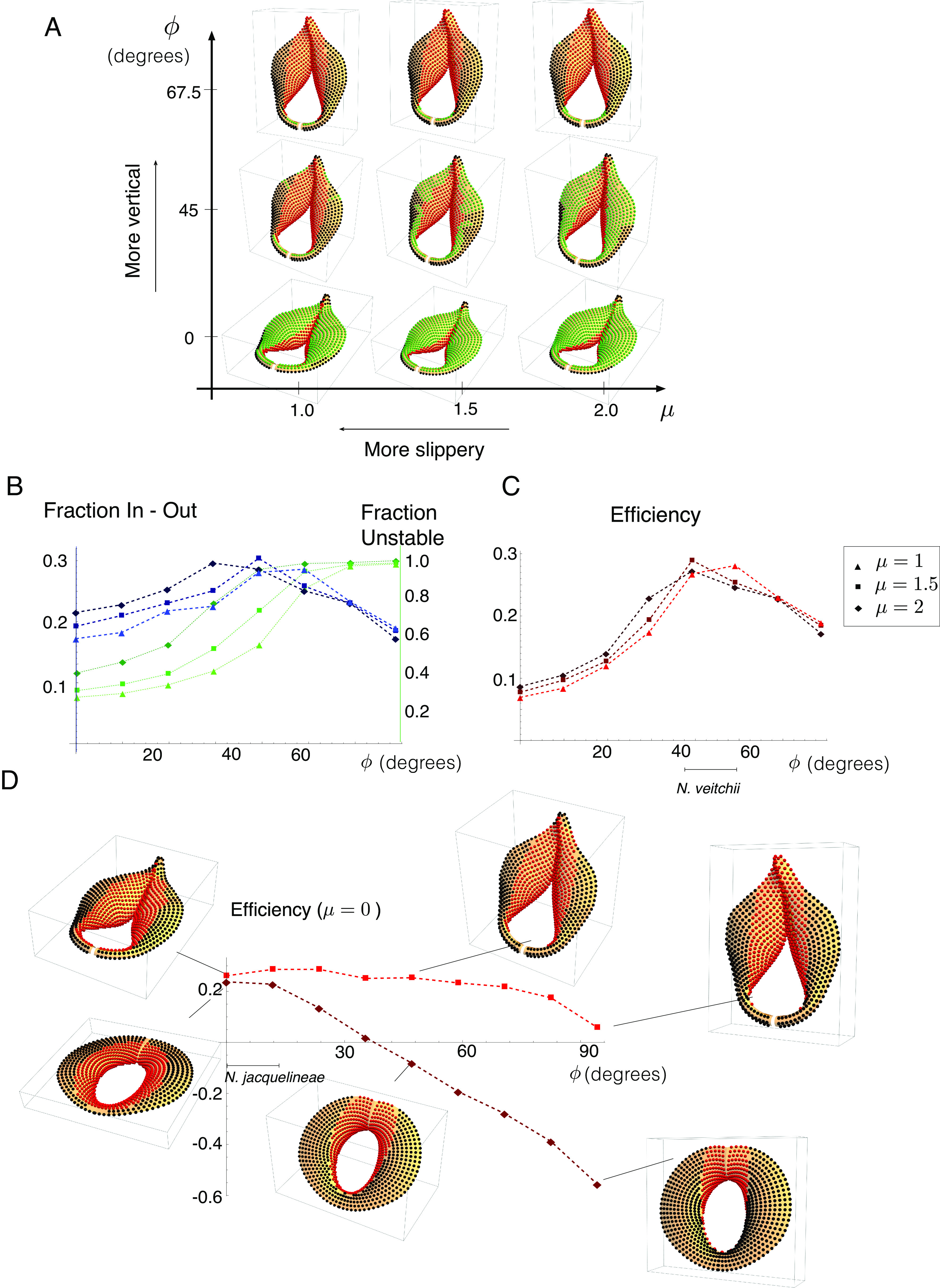
The impact of peristome orientation on prey-capture. (*A*) A phase diagram showing stability and capture properties for varying friction coefficient μ and peristome tilt with respect to the vertical, ϕ, for a model of a flared peristome. Green points are stable, red points slide into the pitcher, and black points slide out. (*B*) Plots of $F_{\text{unstable}}$ (green) and $F_{\text{in-out}}$ (blue) as a function of tilt ϕ for the flared peristome, each for varying values of μ, as indicated. (*C*) Capture efficiency measure as a function of ϕ and varying values of μ. Measured orientation for *N. veitchii* is indicated. (*D*) Efficiency measure as a function of tilt for a fully wetted peristome (μ=0) for the flared peristome model (bright red) curve and a model of *N. jacquelineae* (dark red) displaying a less flared and more uniform peristome geometry. Red points slide into the pitcher and black points slide out. Measured orientation for *N. jacquelineae* is indicated.

To determine the relevance of peristome orientation to prey capture, we have varied this angle, defined as ϕ in our construction (*SI Appendix*, section 1), from being flat (ϕ=0) to vertical ($\phi =90^{{}^{\circ}}$), while also varying the friction coefficient μ. Considering again the Flared peristome model, Fig. 4*A* shows how the regions $\Sigma_{\text{stable}}$ (green), $\Sigma_{\text{in}}$ (red), and $\Sigma_{\text{out}}$ (black) vary both with tilt and friction coefficient. This simulation shows that tilt strongly impacts stability: The flat peristome has most points stable, while the highly tilted peristome has most points unstable. Also of note is that at large and small tilts (*Top* and *Bottom* rows), changing μ has almost no impact, while it has a strong impact at the intermediate tilt $\phi =45^{{}^{\circ}}$. This is particularly relevant in the context of scout ants recruiting large numbers of ants to walk on a surface, and the need for a stable corridor from the edge of the peristome to the unstable-and-fall-in zones. This relies on the stability properties changing with wetness, thus this strategy will be most successful at an intermediate tilt.

To quantify the benefit of a given orientation, we define the following metrics:

[4] $$\begin{array}{c} F_{\text{unstable}}=1-\frac{A_{\text{stable}}}{A}, \end{array}$$

[5] $$\begin{array}{c} F_{\text{in-out}}=\frac{A_{\text{in}}-A_{\text{out}}}{A}, \end{array}$$

where $F_{\text{unstable}}$ is the fraction of the surface that is unstable, while $F_{\text{in-out}}$ is the difference between the fraction of the surface that is unstable and for which dynamic motion leads to falling in and the unstable fraction for which dynamics leads to falling out. These are computed for the Flared peristome in Fig. 4*B*, with $F_{\text{unstable}}$ and $F_{\text{in-out}}$ plotted as green and blues lines respectively, each for three different values of μ. The unstable fraction increases monotonically, such that almost the entire surface is unstable at the vertical orientation $\phi =90^{{}^{\circ}}$, while $F_{\text{in-out}}$ shows a nonmonotonic relation with tilt.

From these metrics, we then compute an efficiency $E:=F_{\text{unstable}}\times F_{\text{in-out}},$ defined as the product of unstable fraction and “in minus out” fraction. A surface with perfect efficiency E=1 is such that every point falls in. Note that with this definition, negative efficiency is possible when more points fall out than in. The efficiency metric is plotted in Fig. 4*C*, and, interestingly, we see that E has a maximum value near $\phi =45^{{}^{\circ}}$ for all values of the friction coefficient, the same range as the tilt we have extracted from *N. veitchii*.

Nevertheless, as noted above, not all species exhibit an approximate 45${}^{{}^{\circ}}$ tilt. For instance, the peristome of *N. jacquelineae* is oriented much closer to the horizontal (ϕ=0 in our description). The peristome of *N. jacquelineae* is also distinctly different from that of *N. veitchii*, with a flatter and more uniform shape, and only a slight gradient toward the center. In Fig. 4*D*, we plot the efficiency metric against ϕ for our model of *N. jacquelineae*. Since the peristome is flat, the surface must become very slippery for any points to become unstable; for this calculation, then, we have set the friction coefficient to zero, so that all points on the surface are unstable. For reference, we also include the same calculation for our model of the Flared peristome. Plotted on this scale, and for a completely slippery surface, the efficiency is nearly constant for *N. veitchii*, showing only a noticeable decrease at the highest tilt. The efficiency of *N. jacquelineae*, on the other hand, decreases significantly and monotonically with increasing tilt, reaching negative values before $\phi =45^{{}^{\circ}}$ and with nearly 60% more points falling out than in at vertical. Because the peristome shape is flat, it requires significant wetting to capture any prey. However, the slight gradient in the geometry is best suited for capture with zero tilt; as the peristome tilt increases, a greater number of points slide off the bottom, rather than being guided inward for capture.

Our model thus predicts a strong link between tilt and prey capture, but in a nontrivial way, with the optimal tilt itself being a function of the peristome shape. Taken together, these results indicate that tilting may be an adaptation to optimize prey capture efficiency.

## 4. On Ribs and Teeth

**Fig. 5.**
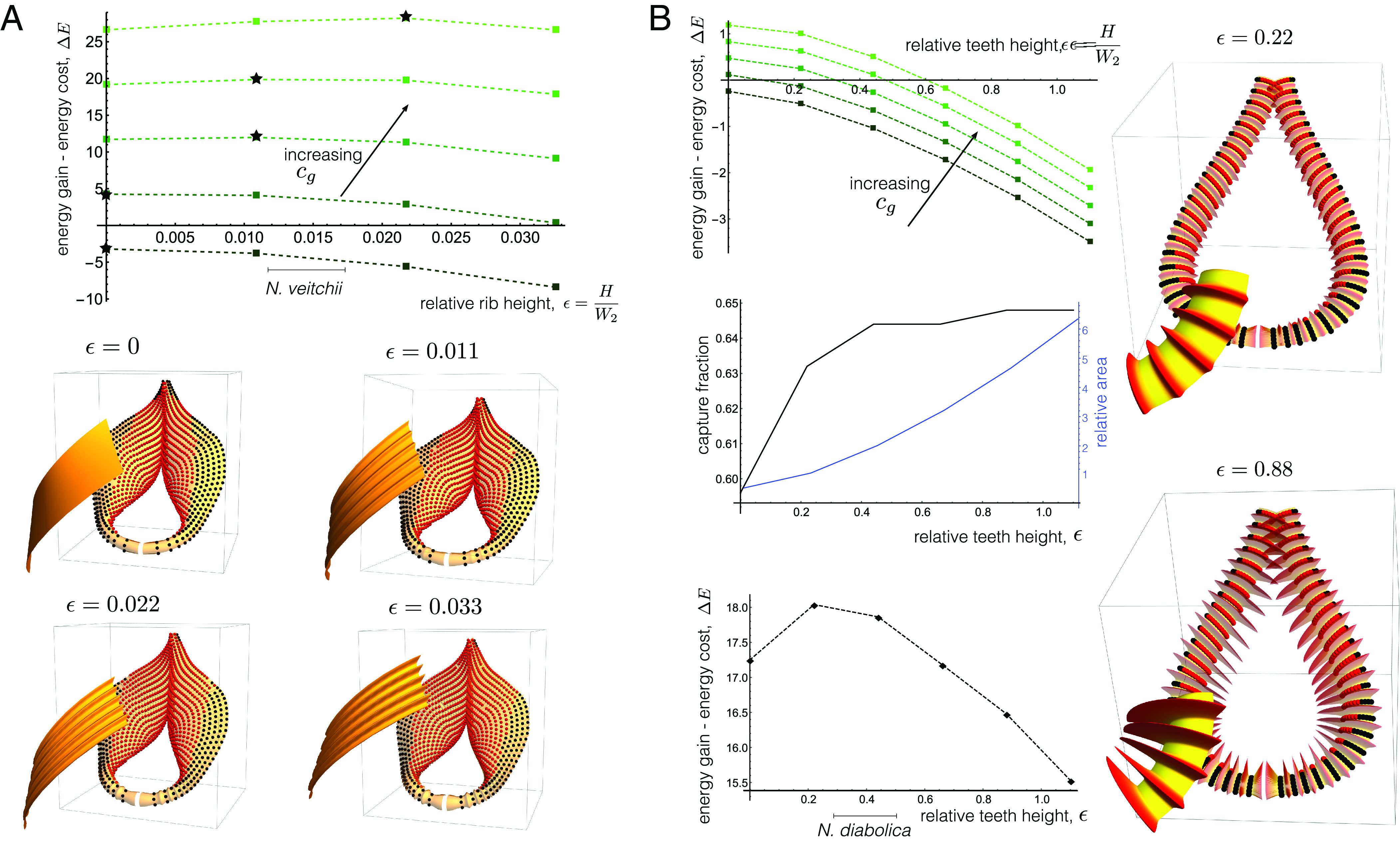
Impact of ribbing and teeth features on prey capture. (*A*) Net energy ΔE plotted against relative rib height for a model of a flared peristome. Energy parameters are $\beta_{g}=\beta_{c}=c_{c}=1$, with $c_{g}$ varying from 1 to 5. Optimal ΔE is indicated with a star. Measured ribbing height range for *N. veitchii* indicated. Below: capture properties for a perfectly wetted surface (μ=0); red points slide into the pitcher, and black points slide out. Zoom in of surface ribbing shown for each height. (*B*) Net energy ΔE plotted against rib/teeth height for a model of *N. diabolica*. Energy parameters are the same as in (*A*) for the *Top* graph. The *Middle* graph plots both the fraction of the surface on which prey are captured, and the surface area relative to a smooth surface. The *Bottom* graph has the energetic gain coefficient $c_{g}$ increased to 50, with other parameters the same. Measured ribbing height range for *N. diabolica* indicated. *Right*: capture properties for perfectly wetted surfaces (μ=0); and zoom in to show teeth features at the indicated heights.

All peristome surfaces possess ribs of varying height and wavelength. In a handful of species, these ribs are highly conspicuous and tooth-like, e.g., in *N. macrophylla*, *N. diabolica* [Fig. 2*D*], *N. villosa* and *N. hamata* (not shown). Phylogenomic data indicate that this phenomenon has evolved independently in the genus *Nepenthes* (15). In this section, we examine the prey-capture benefit that may be obtained from such features, in the context of a cost–benefit analysis. Typically, ribs have sharp peaks and wider smooth valleys. Intuitively, the presence of ribs is beneficial as prey that may have slid off the external pitcher are instead guided into the trap. However, such features increase the area at a potentially substantial energetic cost. Following Section 2, we quantify the energetic cost and benefit trade-off using Eqs. **1** and **2** to define the energetic gain $E_{\text{gain}}$ in terms of capture area, and energetic cost $E_{\text{cost}}$ in terms of total surface area. As before, these are functions of the total surface area (cost) and surface area for which prey slide into the pitcher (gain); for the former, as ribbing features are incorporated into the surface parameterization, we can easily compute the additional area (*SI Appendix*, sections 2 *A* and *C*). The metric of relevance is the net energy $\Delta E=E_{\text{gain}}-E_{\text{cost}}$ [Eq. **3**]. Here, we examine ΔE as a function of a single parameter characterizing the size of the ribs/teeth (the wavelength is consistent with observations of living material—see *SI Appendix*, section 1). We first consider the presence of ribs within a Flared peristome. In Fig. 5*A*, we vary the relative rib height, $\epsilon :=H/W_{2}$, (using the measures defined in Fig. 2) from ϵ=0 (perfectly smooth) to ϵ=0.033—the measured value for *N. veitchii* is ϵ≈0.015, as indicated on the graph. We have used the same form of energy functions f and g as in Fig. 3*B*, and have varied $c_{g}$ from 1 to 5. For large values of ϵ, the net energy begins to decrease, showing that there is a limit that is reached when the construction cost of increased rib height outweighs the benefit of prey capture.

Comparing Fig. 3*B* and Fig. 5*A*, we note that for similar values of $c_{g}$, the optimal degree of flaring and ribbing are in rough correspondence with the extracted values from *N. veitchii*, i.e., our model predicts optimal levels of flaring and ribbing that are consistent with those observed in nature, adding weight to the hypothesis that these features confer a selective advantage in the capture-versus-construction trade-off.

In Fig. 5*B*, we perform the same analysis for a model of a thin peristome with varying teeth heights (still defined as $\epsilon =H/W_{2}$); in the case of large teeth, these correspond to our model of *N. diabolica* [Fig. 2*D*]. The net energy ΔE is plotted against ϵ for the same parameter values as in (*A*). For these values, ΔE decreases monotonically with ϵ and there is no net energetic benefit associated with producing teeth. The *Middle* plot shows both the fraction of seeded points captured (black) and the total surface area divided by the smooth area with ϵ=0 (blue). While teeth do increase the capture fraction, it is only by a small margin, while the area increases by a factor of 6 over the range considered. In other words, the cost significantly outweighs the benefit. Since the construction cost is considerable, it is possible that teeth serve a function that falls outside the scope of our model, for instance, retention of prey. The ends of the teeth project markedly into the interior pitcher and could form a barricade that could prevent large prey from escaping. We should note that the presence of such a prominent feature can be predicted in our framework, but only if the energetic gain of any increased capture is weighted highly. The *Bottom* graph in Fig. 5*B* plots ΔE with $c_{g}$ increased from 1 to 50. Here, an interior maximum occurs at ϵ=0.44, a teeth height similar to the extracted value for *N. diabolica*, indicated, though we stress a 50-fold increase was required in the energetic gain parameter $c_{g}$.

## 5. On Peristome Size

Finally, we explore the effect of peristome size on the efficiency of prey capture. Peristome dimensions vary across the genus, which could be a consequence of divergent selective pressures from differences in prey size and availability.

**Fig. 6.**
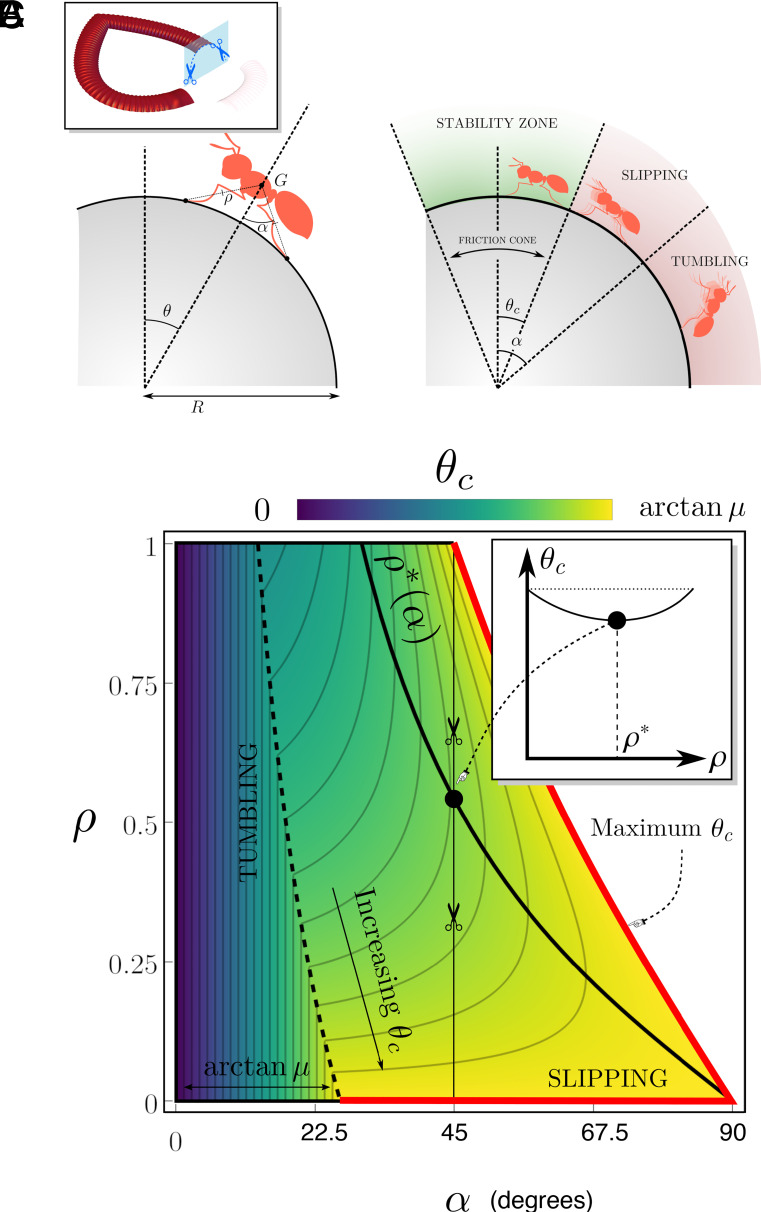
Finite-size prey. (*A*) Schematic of the two-leg prey model geometry. (*B*) The two modes of capture: slipping and tumbling. The friction cone with angle $\theta_{c}$ characterizes the zone where frictional stability can be maintained for a given prey and friction coefficient μ. (*C*) Density plot showing the size of the stability zone ($\theta_{c}$) vs prey angle (α) and prey size (ρ), with μ=0.5. Inset: Plot of $\theta_{c}$ vs ρ. for $\alpha =45^{{}^{\circ}}$. Note that $\theta_{c}\left(\rho \right)$ has a minimum $\theta_{c}^{*}$, reached at a finite value $\rho^{*}$.

The point-mass model is scale-free. Thus, in order to investigate the specific effect of prey size, we consider a minimal representation of a prey with finite size, sitting on a cross-section of a peristome. The peristome is modeled as a circle in a vertical plane, with radius R≡1, taken to be a reference length. The prey is modeled as a rigid body in contact with the peristome at two points located at the same distance ρ from the rigid body’s center of mass G, and with angle 2α between G and the two contact points [Fig. 6*A*]. The scaled length ρ defines the lengthscale of the prey, while α characterizes its shape (flatter insects have larger α). The position of the prey on the peristome is given by $\theta \in [0,45^{{}^{\circ}}]$, the angle between the vertical axis and the prey axis. We assume that the prey is only subject to its own weight, applied at G. As before, we consider dry friction between the prey and the peristome, with coefficient μ at both contact points, and we derive the critical angle for stability, see *SI Appendix*, section 3, and refs. 18 and 19. For each value of ρ and α, and for a fixed friction coefficient μ, we compute exactly the maximum angle $\theta_{c}\leq \alpha$ beyond which equilibrium is lost, and the prey falls. More precisely, for $\theta_{c}<\alpha$, the prey will slip when $\theta =\theta_{c}$, whereas if $\theta_{c}=\alpha$ the prey will lose contact and tumble into the trap. The result is plotted in Fig. 6*C*, where $\theta_{c}$ appears as a color map in the α-ρ plane. A region in which tumbling occurs is indicated on the *Left* side of the plot, for small α. The uncolored white region corresponds to disregarded points in which the leg axis would have to penetrate the surface.

From the perspective of a prey, Fig. 6*C* shows that it is advantageous to be as flat as possible, in the sense that for any ρ, the largest stability angle is achieved when α is maximal. It is also generally the case that very small or very large prey have an advantage. Indeed, for any α, the stability zone $\theta_{c}$ is maximal at the *Bottom* and *Right* edge of the domain (red solid line). This also shows that the point-mass model, which corresponds to ρ→0, provides a lower bound for the trapping efficiency. The function $\theta_{c}\left(\rho \right)$ is nonmonotonic, achieving a minimum value at an intermediate size ρ, denoted $\rho^{*}$ (an exact expression is provided in *SI Appendix*, section 3). This is evident from the valley indicated by the black solid line in Fig. 6*C* and from the inset which plots $\theta_{c}$ against ρ for $\alpha =45^{{}^{\circ}}$. Since we have scaled the insect length by the peristome size, the prey size is r=ρR. Therefore, there is an optimal peristome size $R^{*}\left(\alpha \right)=r/\rho^{*}\left(\alpha \right)$ that will be most effective at capturing the prey. Note that, in the slipping regime, the optimal size $\rho^{*}$ is independent of μ and is, therefore, a universal geometric property of the model. For instance, considering an environment where typical prey have angle $\alpha =45^{{}^{\circ}}$ and size r, and small (but arbitrary) friction coefficient μ≪1, we have $\rho^{*}\approx 0.5$, and the highest trapping efficiency will be achieved by peristomes with R≈2r, which generates a 17% efficiency gain with respect to the most stable case ρ→0, all other things being equal. From an evolutionary viewpoint, this observation suggests the existence of a linear scaling law between the peristome size and the typical size of the prey that will be most easily caught in a particular ecological niche.

A few studies (20, 21) have classified prey contents for a range of *Nepenthes* species in a given habitat, and these seem to be consistent with a correlation between larger peristomes and larger prey, e.g., pitchers with small peristomes, on the order of R≈1 mm in (*N. albomarginata* and *N. gracilis*) almost exclusively captured termites and ants, respectively, while pitchers with larger peristomes, on the order of R≈5 to 10 mm or more (e.g., *N. rafflesiana*) also captured ants, but also captured a wider variety of other prey, including Gasteropoda, Coleoptera, and Arachnida. However, these data do not include measurement of the actual size of the prey trapped and the trend is therefore only qualitative. Moreover, we note that the scaling law we have derived only considers peristomes with constant curvature in which the prey only slips in the plane of the curve. This approximation should be valid in the case of Flat geometries, or Base geometries if the tilting is low enough and/or the ribs are high enough so that slippage occurs in the cross-sectional plane, but is not sufficient to address peristome geometries with significant tilting or flaring. In particular, the larger species in the studies above (*N. rafflesiana* and *N. hemsleyana*) tend to have more curvature variation, and thus slipping can occur in different directions at different points on the peristome.

## 6. Discussion

The remarkable diversity of trap forms in the genus *Nepenthes* is emerging as an adaptive radiation analogous to better-known examples from the animal kingdom, such as the beaks of Darwin’s finches (3). However, the drivers of the adaptive radiation in *Nepenthes* remain poorly known or unexamined in most species. Indeed, little or nothing is known of the prey captured by most of the ca. 200 known *Nepenthes* species. The 30 to 40 species from Borneo are among the best-studied and reveal a diversity of pitcher and peristome morphologies, and prey (22). However, the relationships between these divergent structures, and the prey they trap, remain unclear. By using mathematical modeling and the laws of Newtonian mechanics, our study has provided a theoretical basis for how prey capture may be influenced both by peristome shape and relative size. The diversity of peristomes in *Nepenthes* appears to have evolved in response to dietary needs, adding weight to the hypothesis that a divergence in trap form represents an adaptive radiation.

Carnivory evolved independently in five orders of flowering plants in response to nutrient stress. Advances in genome and transcriptome sequencing have revealed that the repurposing of defense-related genes is an important trend in the evolution of plant carnivory (23). *Nepenthes* evolved within a clade that includes snap trap leaves in the genera *Dionaea* and *Aldrovanda*, in which a touch-sensing mechanism allows rapid closure; and flypaper trap leaves which move more slowly, e.g., *Drosera*. Active mechanisms represent geometric and mechanical solutions adapted for specific prey situations; accordingly, a high diversity of trap configurations has evolved across the various niches occupied by carnivorous plants (24). In *Nepenthes*, prey capture relies on insects being attracted to, and sliding off, the wet peristome. Attraction is achieved by nectar and coloration, while sliding is achieved both by the surface properties of the peristome and the peristome geometry. While the surface properties have been well-documented, here, we link geometry and mechanics—in this case rigid body Newtonian mechanics–to prey capture. Just as in active traps, efficacy is underpinned by both geometry and mechanics.

An optimal geometry might be expected to exist to enable passive capture irrespective of insect type or size. However, we find no such evidence of this; on the contrary, our analysis provides a clear context in which we may understand why peristome geometry in *Nepenthes* is divergent. We consider the value of a given peristome feature in terms of cost–benefit: The energetic cost of peristome construction against the energetic gains of prey capture. While cost–benefit depends on biotic variables, we provide a hypothetical framework for investigating this balance. In the case of peristome flaring, our analysis points to a consistent means by which an evolutionary path from narrow to a flared peristome might exist. Moreover, our analysis may also provide an explanation for an evolutionary divergence in peristome geometry. Indeed, a small change in the parameter $c_{g}$, which characterizes the relative energetic gain of increased prey capture, has a strong impact on the optimal flaring, and for some values, the unflared geometry is energetically optimal. As the energy pathways are likely to vary among species, so will the optimal degree of flaring, and in this context, it is not surprising that not all species possess widely flared peristomes. A similar situation exists in the case of ribbing or teeth features which may generally serve to increase the prey capture functionality, albeit at high production costs. Considering peristome orientation with respect to gravity, our analysis also provides a plausible physical explanation for the correlation between geometry and orientation, demonstrating that a wider and more uniform peristome has better capture efficiency when oriented horizontally.

In these examples, capture success was linked to geometric complexities, and a detailed geometric description was needed for which we sacrificed prey complexity in the description. Conversely, we analyzed finite-sized prey with multiple contact points on a simplified, constant curvature surface restricted to two dimensions. Here again, the connection between geometry and prey specifics was evident and we identified a nonlinear relationship between prey geometry and capture efficiency. Taken together, these results suggest a fine-tuning of peristome size to optimize prey capture likelihood for a given shape and size.

The two distinct forms of analysis we have presented each incorporate simplifications in different ways. Amalgamating the approaches, i.e., combining three-dimensional geometries with a detailed description of finite prey possessing multiple surface contact points, would be more powerful; though it poses a significant challenge to do so in a tractable manner. While our analysis focused on the functional benefits of peristome size and geometry, another problem concerns the developmental process underlying a particular functional geometry. Therefore, a complementary direction of future research would be the morphogenesis of the peristome.

The striking divergence of pitcher forms in *Nepenthes* suggests that they should attract different prey across their various habitats. Prey capture is also known to shift with altitude. Many lowland species are attractive to ants, and possess waxy interior pitcher surfaces effective for capturing these insects (9, 25). By contrast, montane species, which tend to have viscoelastic pitcher fluids, are more effective at trapping flying prey (10, 26). Beetles appear to be the most abundant prey for *N. villosa*, a montane species with conspicuous teeth (20). Peristome teeth may play a role in the retention of bulky prey; however, data from other species with prominent teeth are lacking. Different combinations of pitcher surface and fluid properties probably correlate with peristome size and geometry. For example, pitchers without waxy surfaces often produce larger and more inward-sloping peristomes (25). Importantly, diversity in pitcher form is also probably linked to a vicariance driven by the complex geology and geography of Southeast Asia. Clarke and Moran (27) show that patchy distributions with distinct climates may have contributed significantly to a variation in pitcher form. Soil type too is likely to have influenced local plant community structures, generating specific environmental niches to which the various *Nepenthes* species may be locally adapted. These factors are likely to have played an important part in the evolution of peristome size and geometry. Despite its central role in capture, we know virtually nothing about how prey shifts with changes in peristome morphology. Further work would benefit from empirical and observation data on prey capture from across a range of pitcher and peristome forms in different habitats.

Our study provides a mathematical construct for quantitatively linking geometry to prey capture. There are two distinct steps underlying this link: First is the translation of a given peristome surface to a mathematical object (a surface), and second is the analysis of prey capture on that idealized object. With regard to the former, the surface measures we have defined in Fig. 2*E* provide a direct means of quantifying peristome properties that can then be mapped to prey capture success via the second step, a general mechanism through which inter- and intraspecific variation in geometry and prey capture can naturally be studied. This map may be improved in future work by directly incorporating peristome curvature measures, which we have shown to have significant importance in prey capture success. Investigating this link empirically is a crucial next step. Of course, prey capture will also depend on variables beyond geometry, such as coloration and nectar production. Furthermore, pitcher morphology usually varies with plant age (traps produced by young rosettes are distinct from those on mature vines). In principle, our conceptual approach can accommodate the inclusion of such features. This highlights the value of mathematical modeling as an iterative process that can both motivate and adapt to new empirical studies. In conclusion, this approach provides a platform for testing hypotheses on the evolution of nature’s green predators: some of the plant kingdom’s greatest enigmas.

## Supplementary Material

Appendix 01 (PDF)

## Data Availability

*Mathematica* notebooks reproducing model output are available in the public depository: https://ora.ox.ac.uk/objects/uuid:a7bf597d-07ac-49b0-a444-5b3343618c22 (28).
