# Supplementary material for: Mechanics reveals the role of peristome geometry in prey capture in carnivorous pitcher plants (Nepenthes)
Source: Proc Natl Acad Sci U S A. 2023 Sep 7;120(38):e2306268120. doi: 10.1073/pnas.2306268120 (PMC10515166; doi:10.1073/pnas.2306268120)
Supplement: Supplementary file 1 — Appendix 01 (PDF) [file pnas.2306268120.sapp.pdf]

PNAS

# Supplementary Information for

## Mechanics reveals the role of peristome geometry in prey capture in carnivorous pitcher plants (*Nepenthes*)

**D.E. Moulton, H. Oliveri, A. Goriely & C. Thorogood**

**Correspondence: D.E. Moulton (derek.moulton@maths.ox.ac.uk), C. Thorogood (chris.thorogood@obg.ox.ac.uk)**

**This PDF file includes:**

Figs. S1 to S7
SI References

## Contents

**1 Peristome surface construction** **2**
- A General procedure . . . 2
- B Particular surfaces . . . 4
- C Extracting surface measures . . . 4

**2 Point-mass model** **5**
- A Seeding the surface . . . 5
- B Dry friction . . . 8
- C Dynamic motion . . . 9
- D Energy functions . . . 10

**3 Finite-sized prey** **11**
- A General setup . . . 11
- B Mechanics . . . 11
- C Optimal prey size . . . 13

# 1. Peristome surface construction

## A. General procedure.

**Overview** We first outline the procedure used to generate realistic parameterizations of three-dimensional peristome surfaces. This process is shown schematically in Fig. S1, and consists of the following steps:

1. Define a planar curve $(x(s), y(s))$ for the base shape for the peristome.

2. Define an angle of inclination $\phi$ from the horizontal, i.e. $\phi = 0$ corresponds to a perfectly flat peristome, while $\phi = \pi/2$ for a vertical orientation.

3. Define a function $h_2 = h_2(s)$ describing deviation of peristome base shape from planarity. This was used in constructing models of the peristome for which the proximal end is raised relative to the rest of the peristome.

4. Prescribe the cross-sectional shape of the peristome as a 2D curve, defined in polar coordinates by a function $r(\theta)$, at a finite number of points $\{s_1, s_2, s_3, \ldots, s_n\}$.

5. To create a surface from the set of discrete cross-sectional shapes, we create interpolating functions in $s$ from the values of the shape parameters at each point $s_i$, therefore generating a surface parameterized by $(s, \theta)$.

6. Additional features such as ribbing, teeth, or color patterns may then be added on top.

**More details on Step 4** Most of the fine-tuning for surface construction appears in Step 4, creating the cross-sectional shapes. This step involves a number of sub-steps that we outline further here. First, we note that from the parameterization of the base shape, and given the tilt $\phi$, we define the space curve

$$\mathbf{R}(s) = (x(s), \cos\phi\, y(s), \sin\phi\, y(s)). \quad [1]$$

The tangent $(\mathbf{T})$, normal $(\mathbf{N})$ and binormal $(\mathbf{B})$ vectors to this curve may then be defined at each point $s$ as follows:

$$\alpha(s)\mathbf{T}(s) = (x'(s), \cos\phi\, y'(s), \sin\phi\, y'(s)) \quad [2]$$

$$\alpha(s)\mathbf{N}(s) = (y'(s), \cos\phi\, x'(s), \sin\phi\, x'(s)) \quad [3]$$

$$\mathbf{B}(s) = (0, -\sin\phi, \cos\phi), \quad [4]$$

where $\alpha(s)^2 := x'(s)^2 + y'(s)^2$.

In order to create easily manipulated cross-sectional shapes, we first restrict our attention to a class of functions for the shape, such that the precise shape is determined by a small number of parameters that we can fix at each point $s_i$. For most of the peristome surfaces, we have used logarithmic spirals, i.e. in polar coordinates the curves $r(\theta) = r_0 e^{f\theta}$. The parameter $f$ is used to characterize flaring–$f \to 0$ corresponds to an arc of a circle, with radius $r_0$, while a larger value of $f$ produces a more flared curve (increasing radius as $\theta$ increases).[*]

The shape is initially defined in the **N**-**B** plane, from which we then define two rotations: we rotate the curve about the binormal direction by angle $\varphi$, and rotate the curve about the axis normal to the plane by $\Theta$. For example, if $\varphi = 0$ and $\Theta > 0$, the curve is placed in the **N**-**B** plane, rotated by angle $\Theta$ about the tangent **T**.

The domain of each curve is given by $\theta_1 < \theta < \theta_2$, where the $\theta_i$ are also chosen at each point $s_i$. Combining the above, at each point $s_i$ we define the following parameters:

$$\mathcal{S}_i = \{h_{2_i}, r_{0_i}, f_i, \varphi_i, \Theta_i, \theta_{1_i}, \theta_{2_i}\}. \qquad [5]$$

**More details on Step 5** For given parameters $\mathcal{S}_i,\ i = 1, \ldots, n$, we create interpolating functions, transforming each of the 7 parameters in $\mathcal{S}_i$ into functions in $s$. For example, we generate the flaring function $f(s)$ as an interpolating function passing through each of $\{(s_1, f_1), (s_2, f_2), \ldots, (s_n, f_n)\}$. From these interpolating functions, we then define the peristome surface $\mathbf{P}(s, \theta)$ as follows:

$$\begin{aligned}\mathbf{P}(s,\theta) = &\mathbf{R}(s) \\ &- r_0(s)e^{f(s)\theta}\sin\varphi(s)\big(\cos\Theta(s)\cos\theta - \sin\Theta(s)\sin\theta\big)\mathbf{T}(s) \\ &+ r_0(s)e^{f(s)\theta}\cos\varphi(s)\big(\cos\Theta(s)\cos\theta - \sin\Theta(s)\sin\theta\big)\mathbf{N}(s) \\ &+ \Big[h_2(s) + r_0(s)e^{f(s)\theta}(\sin\Theta(s)\cos\theta + \cos\Theta(s)\sin\theta) - r_0(s)\exp(f(s)(\pi/2 - \Theta(s)))\Big]\mathbf{B}.\end{aligned} \qquad [6]$$

The final term in this expression shifts the curve so that the baseline curve $\mathbf{R}(s) + h_2\mathbf{B}$ is part of the final surface. We found that this choice produced smoother surfaces that were easier to manipulate to have desired features.

For ease of computation, we exploit the bilateral symmetry inherent in peristome geometry, so that we need only define and perform computations on one half (the right half, say) of the peristome, with all sliding properties assumed to be identical.[†] Surface plots in the main text show both halves, where the left half follows a mirror symmetry with the right half.

Via the process outlined above, having fixed the base shape and orientation, the surface is defined by the $7 \times n$ parameters $\mathcal{S}_i,\ i = 1, \ldots, n$. Typically we have used $n = 6$ points to balance efficiency while maintaining sufficient degrees of freedom to control surface geometry properties. With $n = 6$, the 42 parameters were varied using the *Manipulate* command in Mathematica (1), which provides a graphical user interface that rapidly updates the surface geometry while continuously varying any of the parameters. In this way, by monitoring the surface shape and comparing with images of actual *Nepenthes*, parameter sets for the $\mathcal{S}_i$ were constructed for each of the model peristome surfaces.

**Continuous flaring** In generating Fig. 2 of the main text, we have continuously varied the peristome flaring. To do this, we first defined a flaring function $f(s)$, as well as all other surface parameters. We then incorporate a scaling factor $\beta$, so that the flaring function is given by $\beta f(s)$; ranging $\beta$ produces a naturally varying flare while maintaining other surface properties unchanged.

[*] Note that in our construction, the positive $x$-axis for each cross-section is aligned with the normal vector, which points inside the peristome, thus $\theta$ increases from inside edge to outside edge.

[†] Mirror symmetry with sliding would not be maintained if the peristome were rotated along the bilateral axis; however for the vast majority of *Nepenthes* that we have examined, there is negligible rotation of this type.

**On surface features** The process outlined above produces a smooth surface. Adding surface features such as ribbing or teeth is straightforward, as this can be defined by a variation in the cross-sectional radius as a function of $s$. For instance, to add ridges with wavelength $\omega$ and amplitude $\epsilon$, the term $r_0(s)\mathrm{e}^{f(s)\theta}$ is replaced by $r_0(s)\mathrm{e}^{f(s)\theta} + \epsilon\cos(\omega\lambda(s))^m$ where $m$ is an integer characterizing the sharpness of the ridges ($m = 1$ for perfectly sinusoidal ridges, while a large value of $m$ produces ridges with sharp peaks and wide valleys). The function $\lambda(s)$ is the arc length of the baseline curve, defined by $\lambda'(s) = \alpha(s)$; this term is needed to maintain a constant wavelength, as the base curve is not defined (necessarily) to be an arc length parameterization. Both ridges and teeth may be defined in the same way; the main difference being that with teeth we use a much larger value of both $\epsilon$ and $m$, e.g. $m = 4$ for ridges and $m = 40$ for teeth. Note that in defining ridges and teeth in this way, they are aligned with the cross-sectional curves; therefore the function $\varphi(s)$ is used to rotate this alignment, for instance, to be more oblique towards the proximal end, as we have observed in many species.

Coloration of surfaces in Fig. 1 of the main text was purely added for visual purposes in comparison with real specimens. Color patterns were produced using the *ColorFunction* environment in Mathematica.

**B. Particular surfaces.** Specific values of the parameters and functions used for the peristome models can be found in the deposited Mathematica notebooks, which include the construction of each of the models appearing in main text Fig. 2.

**C. Extracting surface measures.** In main text Fig. 2(e), we have defined 5 surface measures characterizing the peristome properties. These are the interior peristome length ($L$), the minimum ($W_1$) and maximum ($W_2$) peristome widths, the angle of the peristome with respect to gravity ($\phi$), and the average ribbing height ($H$). From these, the peristome relative width is defined as $W_2/L$, the degree of flaring is $W_2/W_1$, the prominence of ribs/teeth is defined to be $H/W_1$, and the orientation is the angle $\phi$. Main text Fig. 2 (e)(ii)-(iii) plots these values for the sample species appearing in (a)-(d), both for the real specimens and the model reconstructions. Our procedure for extracting the values from the real specimens was to overlay straight lines on the images, from which the lengths $L, W_1, W_2$, and $H$ were approximated by the lengths of the lines (in pixels); as the relevant measures are ratios and angles, the actual dimensional lengths were not needed.

We stress that these extracted values are approximations, with varying levels of precision based on the type of measure. For instance, while the lengths and widths could be extracted with reasonable precision, the ribbing features on all but the Toothed category are very small and difficult to measure accurately from a photo. Peristome images with lines overlaid are provided in Figs. S2 and S3.

In terms of the mathematical surface reconstructions, these parameter values are more readily extracted. The length $L$ is computed from the space curve $\mathbf{R}$; in particular by the symmetry of the peristome surface we have $L := y(s_n) - y(s_1)$. In extracting the maximum and minimum peristome widths, we first define the peristome width function $W(s) := ||\mathbf{P}(s, \theta_1(s)) - \mathbf{P}(s, \theta_2(s))||$, that is the norm of the vector pointing from inside to outside edge of the cross-section; we then equate the minimum and maximum values of $W$ as $s$ varies from $s_1$ to $s_n$ with $W_1$ and $W_2$, respectively. The angle $\phi$ and ribbing height $H$ are direct inputs to the mathematical surface so these are immediately accessible.

**Ribbing wavelength** To justify the choice of ribbing wavelength, particularly in the case of main text Fig. 5(a), in Fig. S4 we show side by side an image of *N. veitchii* and a model representation. In each, we have indicated a segment of peristome corresponding to 50 ribs, with every 5th rib indicated by a white circle. This shows that while not exact, the wavelength we have used in our model is in rough agreement with the wavelength in real specimens.

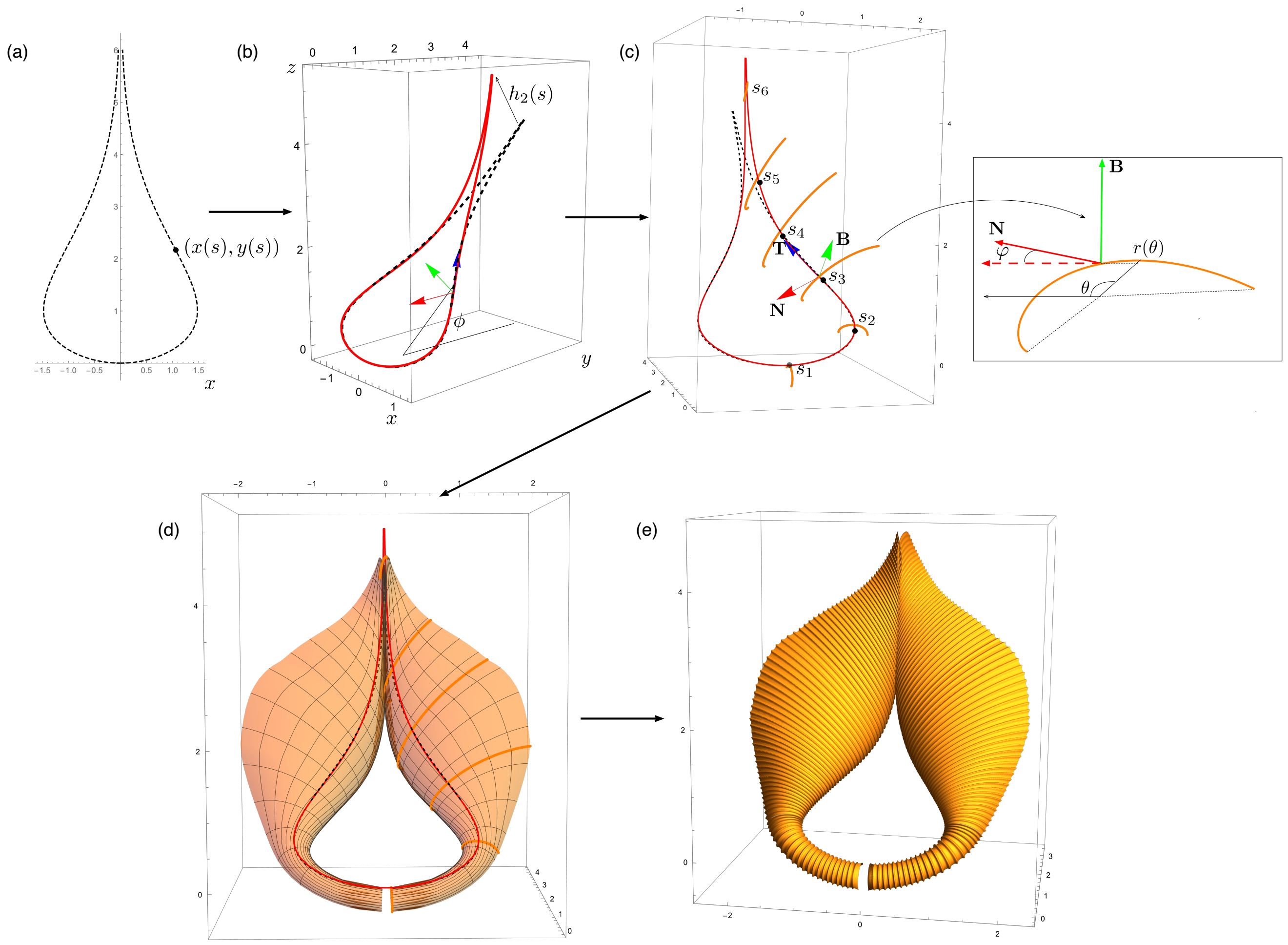


**Fig. S1.** Peristome surface construction. (a) A plane curve $(x(s), y(s))$ is constructed, and (b) translated out of plane by an amount $h_2(s)$ and rotated by angle $\phi$ about the $x$-axis, with the tangent-normal-binormal frame (TNB) is defined at each point on the curve. (c) At a discrete set of points $\{s_1, s_2, \dots, s_n\}$, a cross-sectional curve is defined, originally in the normal-binormal plane, but rotated by angle $\varphi$ about the binormal. (d) a surface is created by interpolating the functions defining the discrete cross-sectional shapes. (e) additional features such as ribbing are added.

## 2. Point-mass model

Here we outline the procedure for stability properties and dynamic motion of a point mass on the surfaces as constructed above.

**A. Seeding the surface.** While some properties, such as stability under dry friction, can be readily computed as a continuum property at each point on the surface, computation of the dynamics requires integrating the equations of motion forward in time, which must be done individually for any given point on the surface. Therefore, in order to approximate the relative areas of the surface for each category–stable, unstable with dynamics leading to falling into the pitcher, and unstable with dynamics leading to falling out of the pitcher–we first seed the surface with a large number of points. In order to space the seed points evenly across the surface, we first compute the metric tensor $\mathbf{G}$, which has components

$$G_{11} = \frac{\partial \mathbf{P}}{\partial s} \cdot \frac{\partial \mathbf{P}}{\partial s}, \quad G_{12} = \frac{\partial \mathbf{P}}{\partial s} \cdot \frac{\partial \mathbf{P}}{\partial \theta}, \quad G_{22} = \frac{\partial \mathbf{P}}{\partial \theta} \cdot \frac{\partial \mathbf{P}}{\partial \theta}. \qquad [7]$$

Noting that the line element $\mathrm{d}S$ of a curve on the surface satisfies $\mathrm{d}S^2 = G_{11}\mathrm{d}s^2 + 2G_{12}\mathrm{d}s\mathrm{d}\theta + G_{22}\mathrm{d}\theta^2$, our approach for seeding the surface is then as follows:

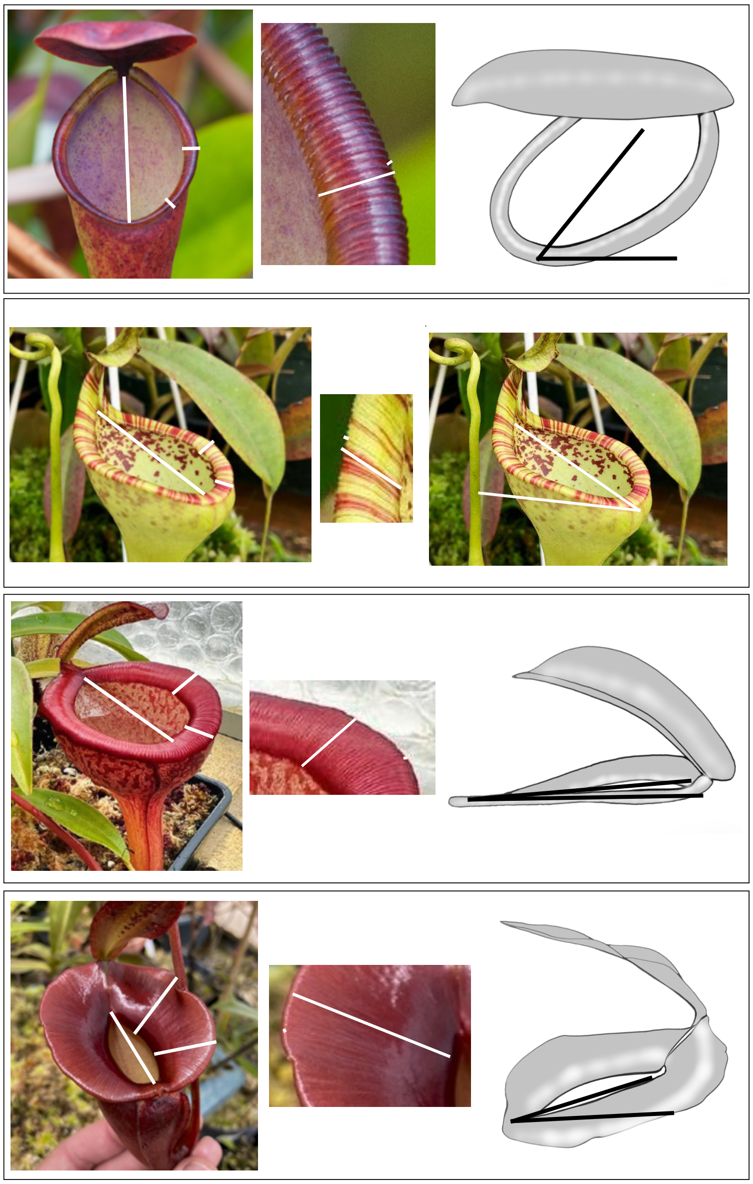

**Fig. S2.** Peristome images and overlay of lines from which we have extracted the dimensionless surface measures $L$, $W_1$ and $W_2$ (left), $H$ and $W_2$ (middle), and $\phi$ (right) for the species (top to bottom): *N. pervillei*, *N. eymae*, *N. jamban*, and *N. jacquelinae*.

1. Fix a step size $\delta$
2. Fix $s = s_1$ (distal end).
3. Seed points along the cross-section:
   (i) Fix $\theta = \theta_1(s)$, and seed a point
   (ii) Increment $\theta \leftarrow \theta + \frac{\delta}{\sqrt{G_{22}(s,\theta)}}$, and seed a point
   (iii) Continue until $\theta_2$ is reached.
4. Increment $s \leftarrow s + \frac{\delta}{\sqrt{G_{11}(s,\theta)}}$

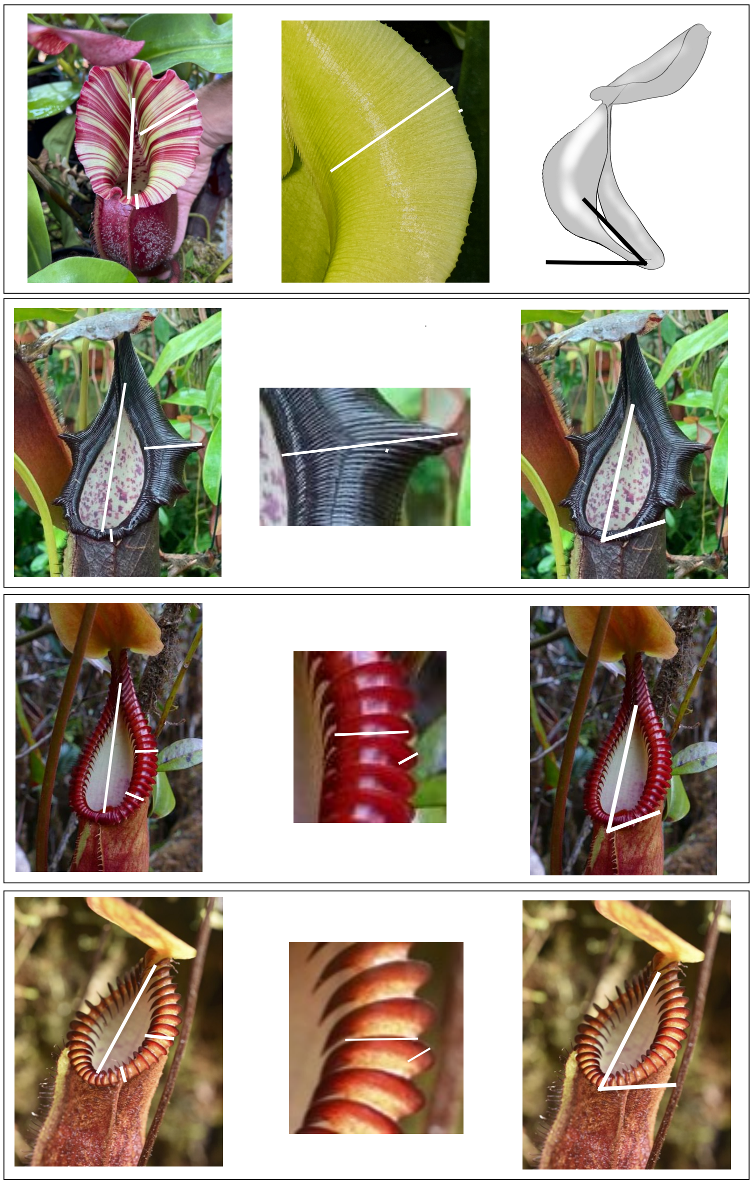

**Fig. S3.** Peristome images and overlay of lines from which we have extracted the dimensionless surface measures $L$, $W_1$ and $W_2$ (left), $H$ and $W_2$ (middle), and $\phi$ (right) for the species (top to bottom): *N. veitchii*, *N. naga*, *N. macrophylla*, and *N. diabolica*.

5. Return to step 3.

6. Repeat until $s \geq s_n$ is reached.

In the case of ribbing/teeth, in producing main text Fig. 5 we only seed points in the valleys of the surface features. That is, given the cross-sectional shape $r_0(s)e^{f(s)\theta} + \epsilon\cos(\omega\lambda(s))^m$, we seed points at values of $s$ satisfying $\omega\lambda(s) = (2n+1)\pi/2$ for integers $n$. This produces the same uniform seeding for all values of $\epsilon$, since $\omega$ and $\lambda s$ are fixed for a given peristome shape, and ensures that the starting points for the dynamics are treated equally for all ribbing/teeth heights (the dynamic motion will be much different for a point starting on the top of a ribbing feature). Intuitively, in the case of large teeth features, as e.g. on *N. diabolica*, flying prey are very unlikely to land on the top or side of a tooth feature, and also walking prey seem most likely to enter the surface in the valleys, though it remains to investigate this empirically.

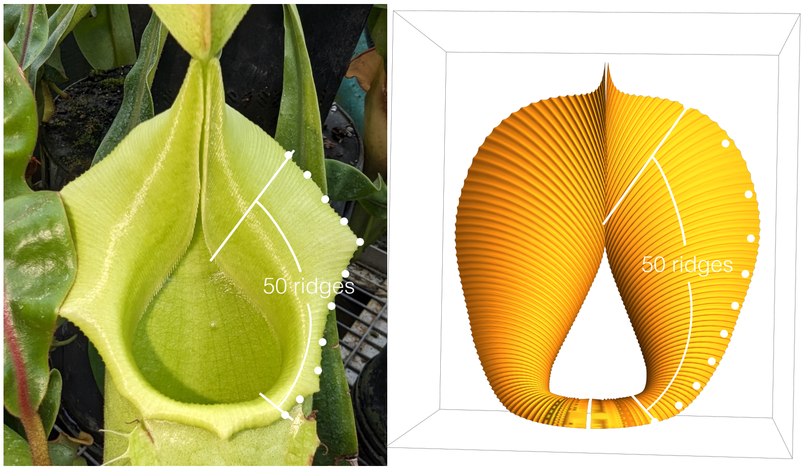


**Fig. S4.** Ribs count on *N. veitchii* and a model representation, with every 5th rib marked and a region corresponding to 50 ribs indicated.

**B. Dry friction.** The criterion for stability under dry friction is

$$\frac{F}{N} < \mu, \tag{8}$$

where $F$ and $N$ are the components of the reaction force $\mathbf{r}$ tangential to and normal to the surface, respectively, and $\mu$ is the friction coefficient (2). As the only force acting on the point mass is gravity, which is defined in the $z$-direction (and can be defined with unit magnitude without loss of generality), the reaction force satisfies $\mathbf{r} = \mathbf{e}_z$. The component of $\mathbf{r}$ normal to the surface has magnitude $N = |\mathbf{e}_z \cdot \mathbf{n}|$, where $\mathbf{n}$ is a unit normal vector, which can be computed via

$$\mathbf{n} = \frac{\frac{\partial \mathbf{P}}{\partial s} \times \frac{\partial \mathbf{P}}{\partial \theta}}{\left\| \frac{\partial \mathbf{P}}{\partial s} \times \frac{\partial \mathbf{P}}{\partial \theta} \right\|}. \tag{9}$$

The frictional component of $\mathbf{r}$ is then $\mathbf{e}_z - n\mathbf{N}$, from which we determine that the point is stable if [Eq. (8)]

$$\frac{F}{N} = \frac{\sqrt{1 - (\mathbf{e}_z \cdot \mathbf{n})^2}}{|\mathbf{e}_z \cdot \mathbf{n}|} < \mu. \tag{10}$$

**Computational aside** It is worthwhile to note that the expressions for $G_{ij}$ as well as $\mathbf{n}$, while straightforward to compute, are very long for the surface defined by Eq. (6), and can be cumbersome to apply. For computational ease, it is useful to take advantage of the orthonormality of the right-handed basis $\{\mathbf{T}, \mathbf{N}, \mathbf{B}\}$, which satisfies the Frenet equations

$$\begin{aligned} \mathbf{T}'(s) &= \alpha(s)\kappa(s)\mathbf{N} \\ \mathbf{N}'(s) &= -\alpha(s)\kappa(s)\mathbf{T} \\ \mathbf{B}'(s) &= 0, \end{aligned} \tag{11}$$

where the curvature $\kappa$ is given by

$$\kappa(s) = \frac{x'(s)y''(s) - x''(s)y'(s)}{\alpha(s)^3}.$$

Since the surface $\mathbf{P}$ is expressed in this basis, the scalar and vector products of the partial derivatives with respect to $s$ and $\theta$ take a reduced form when all derivatives are expressed in the basis itself using Eq. (11). Our approach was to pre-compute generic analytical expressions for the $G_{ij}$ and $\mathbf{n}$ from Eq. (6) before explicitly defining the interpolating functions. Specific computed formulas are available in the Mathematica notebooks accompanying this manuscript.

### C. Dynamic motion.

**Equations of motion** The equations of motion for a point mass sliding on the surface are determined via the Euler-Lagrange equations with energy function having components for both kinetic energy and gravitational potential energy[‡]. Noting that the path along the surface is defined by a curve in parameter space $(s(t), \theta(t))$, the position vector for the material point is given by $\mathbf{p}(t) = \mathbf{P}(s(t), \theta(t))$. The kinetic energy for mass $m$ then takes the usual form

$$\mathcal{T} = \frac{1}{2} m |\dot{\mathbf{p}}|^2, \tag{12}$$

where overdot denotes a material derivative with respect to time, i.e. $\dot{\mathbf{p}}(t) = \dot{s}(t)\, \partial \mathbf{P}/\partial s(s(t), \theta(t)) + \dot{\theta}(t)\, \partial \mathbf{P}/\partial \theta(s(t), \theta(t))$, while the potential energy is given by

$$\mathcal{V} = mg\mathbf{p} \cdot \mathbf{e}_z, \tag{13}$$

with $g$ the gravity of Earth. Each of these may be expressed in terms of the functions $s(t)$ and $\theta(t)$ via the parameterization given by Eq. (6), from which we may write the Lagrangian $\mathcal{L} = \mathcal{T} - \mathcal{V}$:

$$\mathcal{L}(s(t), \theta(t), \dot{s}(t), \dot{\theta}(t)) = \frac{1}{2} m |\dot{\mathbf{p}}(t)|^2 - mg\mathbf{p}(t) \cdot \mathbf{e}_z. \tag{14}$$

The equations of motion are then given by

$$\frac{\mathrm{d}}{\mathrm{dt}} \frac{\partial \mathcal{L}}{\partial \dot{s}} - \frac{\partial \mathcal{L}}{\partial s} = 0, \tag{15}$$

$$\frac{\mathrm{d}}{\mathrm{dt}} \frac{\partial \mathcal{L}}{\partial \dot{\theta}} - \frac{\partial \mathcal{L}}{\partial \theta} = 0. \tag{16}$$

As initial conditions, we prescribe zero initial velocity and choose $(s(0), \theta(0))$ to correspond to the values of seed points as described above. Eqs. (15) and (16) define two highly nonlinear coupled second order differential equations for $(s(t), \theta(t))$. Again, the calculation is straightforward; the challenge is in dealing efficiently with the cumbersome formulas. As above, this is rendered easier by expressing all derivatives back in terms of the orthonormal basis $\{\mathbf{T}, \mathbf{N}, \mathbf{B}\}$. Having reduced the formulas as outlined, the system of equations can generally be integrated forward in time in less than a second for points with simple motions (e.g. a point near the edge), or maximally within tens of seconds for more complicated motions (e.g. a point situated next to a large tooth). These were integrated using the numerical solver *NDSolve* in Mathematica, with a stopping criterion based on reaching the edge of the surface. In particular, if $\theta(t)$ reaches $\theta_1$, the point has reached the inside edge, and we deem the mass to have fallen in the pitcher; conversely, if $\theta(t)$ reaches $\theta_2$, the point has reached the outside edge, and we deem the mass to have fallen out of the pitcher. Given the mirror symmetry, it is also possible for the point to reach the proximal point, defined by $s(t)$ reaching 0; in this event, we simply reflect the velocities as appropriate and continue the integration.

The gravitational constant $g$ only has the effect of increasing or decreasing the rate with which the mass slides off the surface, and could be set arbitrarily. Here, we note that when computing the dynamic motion, we assume that the surface is fully wetted. In this case, every point is unstable except for points at which the surface is flat with respect to gravity and with appropriate sign of the mean curvature, i.e. a point situated at a local minimum. For the geometries we considered, this was not an issue, i.e. there were no attracting regions on the surface into which a point could slide and potentially remain. If such regions did exist, the value of the gravitational constant would be important in determining the velocity with which a point entered such a region and therefore whether it became stuck; but this was not the case in our simulations.

[‡] For simplicity we neglect kinetic friction, which should be largely irrelevant with regards to the question of whether the point mass slides into or out of the pitcher.

**Fall in/fall out percentages and surface area** Having computed the dynamic trajectories of each of the seed points, we can approximate the percentage of the surface area for which dynamic motion leads to falling in versus out by simply counting the number of seed points that lead to the different stopping criteria. That is, if the dynamic trajectories of $N_1$ seed points ended with $\theta = \theta_1$ (falling in), and there were $M$ total seed points, we approximate $N_1/M \times 100$ as the percentage of the surface for which prey "fall-in".

In terms of actual surface area, which is relevant to our energy analysis, we may compute the total surface area in the usual way, in terms of the metric tensor:

$$\mathcal{A} = \int_{s_1}^{s_n} \int_{\theta_1(s)}^{\theta_2(s)} \sqrt{\det \mathbf{G}}\, \mathrm{d}\theta \mathrm{d}s. \quad [17]$$

Note that ribbing features do not change the nature of the calculation–as ribbing/teeth are directly incorporated into the surface parameterization, the ribbing pattern and properties (height $\epsilon$ and frequency $\omega$) are built into the metric tensor, so that the formula above provides $\mathcal{A} = \mathcal{A}(\epsilon)$ when increasing the ribbing height as in main text Fig. 5. Note that in terms of energetic gain, because we only seed points in the valleys (as described in Section 2A above), we compute the capture area as the percentage of seed points that fall into the pitcher times the area of the *smooth* surface.

**D. Energy functions.** We have defined in the main text the net energy

$$\Delta E = E_{\text{gain}} - E_{\text{cost}} = g(\mathcal{A}_{\text{in}}) - f(\mathcal{A}), \quad [18]$$

where $\mathcal{A}_{\text{in}}$ is the surface area for which prey will fall into the peristome, $\mathcal{A}$ is the total surface area, and $f$ and $g$ are functions that model respectively the link between prey capture and energetic benefit for the plant, and the energetic cost of constructing and maintaining the peristome. We have considered the functional forms $g(x) = c_g x^{\beta_g}$, $f(x) = c_c x^{\beta_c}$, where the constants $c_g$ and $c_c$ characterize the energetic gains and costs, respectively, while the exponents $\beta_g$ and $\beta_c$ characterize possible non-linearity in the pathway between areas and energy.

For simplicity, in the results presented in the main text, we only varied $c_g$ with fixed values $c_c = 1$ (a choice that can be made without loss of generality since the absolute energy is not relevant in our analysis), $\beta_g = \beta_c = 1$. In this section we explore in more detail the impact of the $\beta$ parameters, in particular with respect to the potential energetic benefit of flaring, corresponding to main text Fig. 3. Fig. S5 plots $\Delta E$ against the flaring parameter $W_2/W_1$ for $\beta_g$ and $\beta_g$ ranging over the discrete set $\{0.2, 1.0, 2.5\}$. These plots show a range of qualitatively different behavior. Consider first the middle column of plots, with $\beta_c = 1$. If $\beta_g < 1$, the energetic gain per area increase is relatively large when the area is small, but decreases as the area increases (since the derivative of the function $x^a$ with $a < 1$ diverges as $x \to 0$ but vanishes as $x \to \infty$). The result is that $\Delta E$ is a monotonically decreasing function of $W_2/W_1$ in the case $(\beta_g, \beta_c) = (0.2, 1.0)$; here it is not energetically favorable to increase flaring. As $\beta_g$ is increased to 1.0 (middle plot), $\Delta E$ has an internal maximum for most values of $c_g$, so that flaring to some degree is favorable. If $\beta_g$ is increased further, the energetic gain per area increase is small when the area is small, but increases with increasing area (the function $x^a$ with $a > 1$ has increasing derivative). The result is an initial decrease of $\Delta E$, followed by a sharp rise in the case $(\beta_g, \beta_c) = (2.5, 1.0)$. This shape has an interesting theoretical evolutionary consequence: even though it is highly energetically favorable to have large flaring, the initial decrease in $\Delta E$ would cause an energetic barrier, barring an energetically beneficial continuous transition from thin to flared peristome.

In the bottom row of Fig. S5, with $\beta_c = 0.2$, $\Delta E$ is monotonically increasing for almost all values of $c_g$ and $\beta_g$. It is clear that $\Delta E$ should rise with larger $W_2/W_1$, since the energetic cost per increased area is very low for large area. For smaller areas, even though the change in cost per increase in area is higher, the capture fraction also increases the most with small flaring (not plotted), so that the gain still outweighs the cost. In the top row, $\beta_c = 2.5$, the energetic cost is very high compared to the gain, and $\Delta E$ is monotonically decreasing, though $\Delta E$ is nearly flat for small flaring, as the cost is less impactful at small areas.

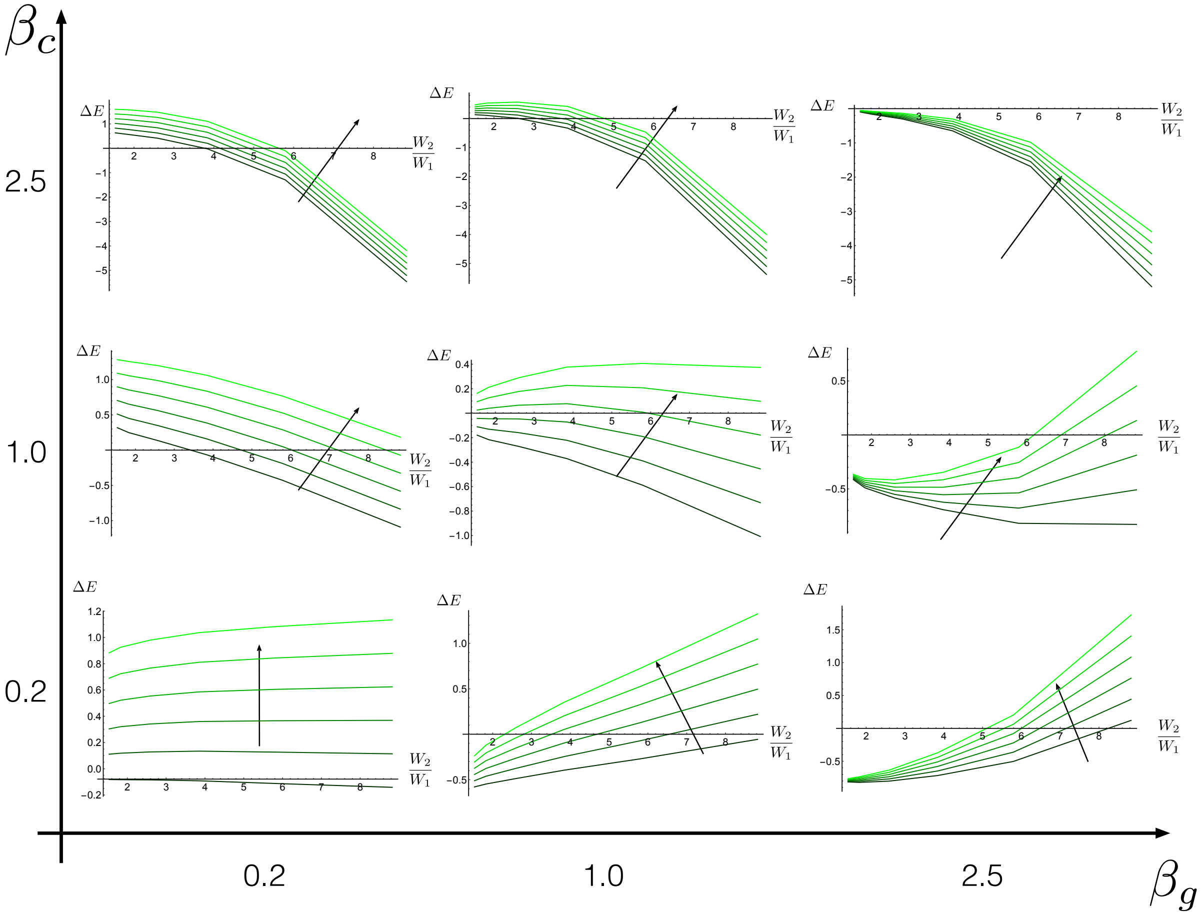


**Fig. S5.** Net energy $\Delta E$ plotted against flaring parameter $\alpha_f$ for varying values of $\beta_g$ and $\beta_c$. Within each plot, arrows indicate increasing $c_g$ from 1 to 2.25.

## 3. Finite-sized prey

We detail the rigid body model used to investigate the effect of prey size.

**A. General setup.** We model the prey as a bilaterally symmetric rigid body with center of mass $G$ and (arbitrary) mass $m$ [Fig. S6(a)]. The body is in contact with the peristome at two points located at the same distance $\rho$ from $G$, respectively noted $C_1$ and $C_2$ (with $C_1$ the highest point). We introduce $\gamma$, the radius of gyration of the rigid body, and the angle $\alpha := 1/2 \times \widehat{C_1GC_2}$. The peristome is modeled as a circle with radius $R \equiv 1$, used as a reference length unit. The prey position is parameterized by the angle $\theta$, the angle between the prey and the vertical. We assume that the prey is subject to its own weight only. Lastly, we also prevent the leg axes from intersecting the surface by enforcing the non-penetration constraint

$$\rho \tan \alpha \leq 1. \qquad [19]$$

**B. Mechanics.** To characterize the frictional stability of the prey, we use Erdmann's theory of Coulombian friction with multiple contact points which extends the classic notion of *friction cone* to some appropriately-defined configuration space (3, 4). Indeed, in contrast to a material point, a rigid body can be subject to both a resultant and a torque applied at $G$, that overall form a *generalized force* $(F_x, F_y, \tau) \in \mathbb{R}^3$. In particular, the *generalized friction cone* is a three-dimensional, polyhedral cone that contains all the possible generalized forces that the surface can provide under static friction while maintaining contact at both

contact points. The theoretical basis of the configuration space friction cone is mathematically sophisticated; we refer the reader to Refs. (3, 4) for details. Here, we adapt the theory to our problem and briefly outline the construction of our slipping criterion. The strategy is as follows: i) construct the individual friction cone associated with each contact point; ii) obtain the composite, generalized friction cone (accounting for both contact points) by the superposition principle.

We here build the friction cone for the first contact point $C_1$ (the second friction cone for $C_2$ is obtained similarly). We first define the position vector $\mathbf{r} := \overrightarrow{C_1G} = (r_x, r_y, 0) = \rho(\sin\alpha, \cos\alpha, 0)$, expressed in the canonical basis $(\mathbf{e}_\theta, \mathbf{e}_r, \mathbf{e}_\theta \times \mathbf{e}_r)$ attached to the rigid body [Fig. S6(a)]. In this basis, the weight is expressed as $\mathbf{F} = mg(\sin\theta, -\cos\theta, 0)$. Elementary geometry also provides the normal to the surface at the contact point:

$$\mathbf{n}_0 = (n_x, n_y, n_z) = \left(-\rho\sin\alpha, \sqrt{1-\rho^2\sin^2\alpha}, 0\right). \quad [20]$$

The generalized normal follows as:

$$\mathbf{n} = \frac{1}{\Delta_n}\left(n_x, n_y, \frac{n_x r_y - n_y r_x}{\gamma}\right), \quad [21]$$

with $\Delta_n = \sqrt{1 + (n_x r_y - n_y r_x)^2/\gamma^2}$, such that $\|\mathbf{n}\| = 1$. We stress that, while $\mathbf{n}_0$ and $\mathbf{n}$ have the same dimension, the former is a vector in the physical space, while the latter lives in the 3D configuration space. The last component of $\mathbf{n}$ corresponds to the torque about the reference point $G$, due to a unit reaction force applied at the contact point. Friction acts along the tangent through the point of contact, associated similarly with the configuration vector

$$\mathbf{v}_f = \left(n_y, -n_x, \frac{n_x r_x + n_y r_y}{\gamma}\right). \quad [22]$$

The friction cone for $C_1$ [blue cone in Fig. S6(b)] can be written as $\{a(\Delta_n\mathbf{n} + s\mu\mathbf{v}_f) \,|\, a \geq 0, s \in [-1, +1]\}$ (the one for $C_2$ is obtained analogously). Physically, the fact that the friction cone has a component in the $\tau$ direction accounts for the fact that, if only one contact point is considered, equilibrium of the rigid body requires a non-zero applied torque to balance the applied resultant that tends to induce rotation about the contact point. Finally, the generalized friction cone, which accounts for both contact points, is obtained by the superposition principle and is given mathematically by the vector sum of the two friction cones associated with all contact points [Fig. S6(b)]. Note that to compute the friction cone, one need not compute explicitly the reaction forces at the contact points.

In our scenario, we consider a prey subject to its own weight, with no applied torque. Therefore, we consider the intersection of the three-dimensional friction cone previously constructed, with the plane $\tau = 0$ that describes the space of resultant forces, i.e. torque-free generalized forces. This intersection is a planar cone [in green in Fig. S6(b)] which, as in the case of a material point, provides the maximum inclination above which slippage occurs. The director lines for this cone [thick dashed lines in Fig. S6(b)] are given by $\{a(s\sin\theta_c, \cos\theta_c, 0) \,|\, a \geq 0, s = \pm 1\}$, where $\theta_c$ denotes the critical slippage angle. Tedious, but straightforward calculation of the intersection provides

$$\theta_c = \arctan\left(\frac{\mu}{1 + \rho\left(\mu^2+1\right)\left(\cos\alpha\sqrt{1-\rho^2\sin^2\alpha} - \sin^2\alpha\right)}\right), \quad [23]$$

for a small enough friction coefficient $\mu \leq \mu_c$, with

$$\mu_c^2 = \frac{\sin\alpha + \rho\sin 2\alpha\sqrt{1-\rho^2\sin^2\alpha} + \rho^2\sin\alpha\cos 2\alpha}{\cos\alpha\cot\alpha - \rho\sin 2\alpha\sqrt{1-\rho^2\sin^2\alpha} - \rho^2\sin\alpha\cos 2\alpha}; \quad [24]$$

otherwise

$$\theta_c = \alpha \quad [25]$$

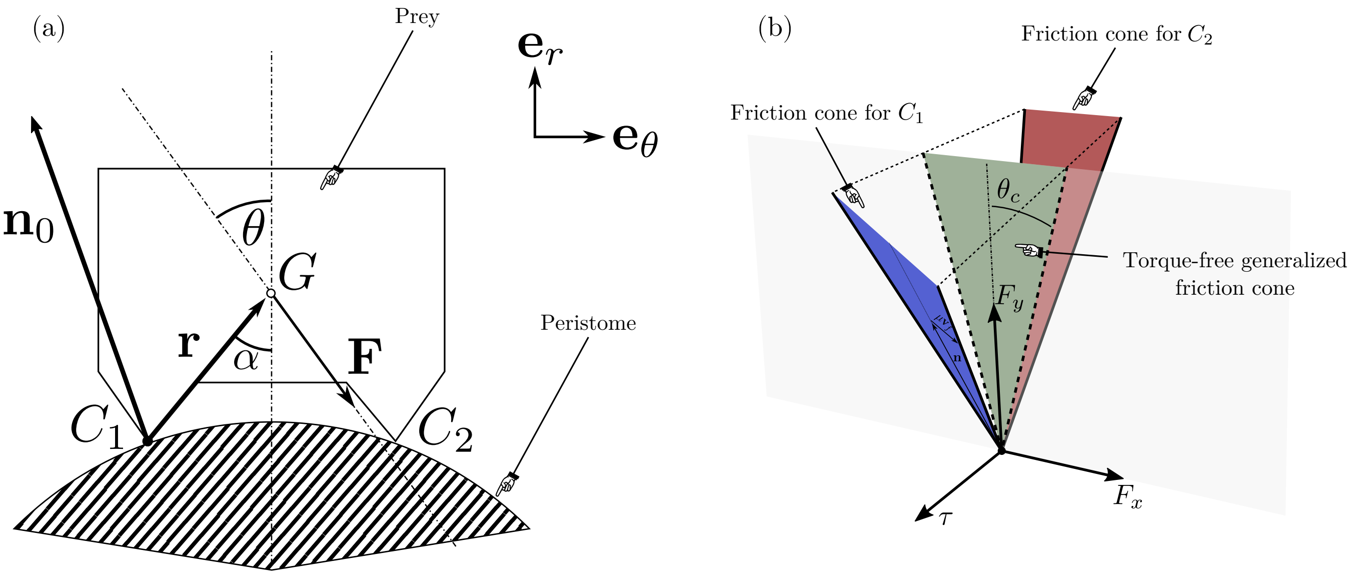


**Fig. S6.** (a) Schematic of a prey modeled as a symmetric rigid body sitting on a circular peristome. (b) The configuration space friction cone intersected with the plane $\tau = 0$ provides the critical inclination angle $\theta_c$.

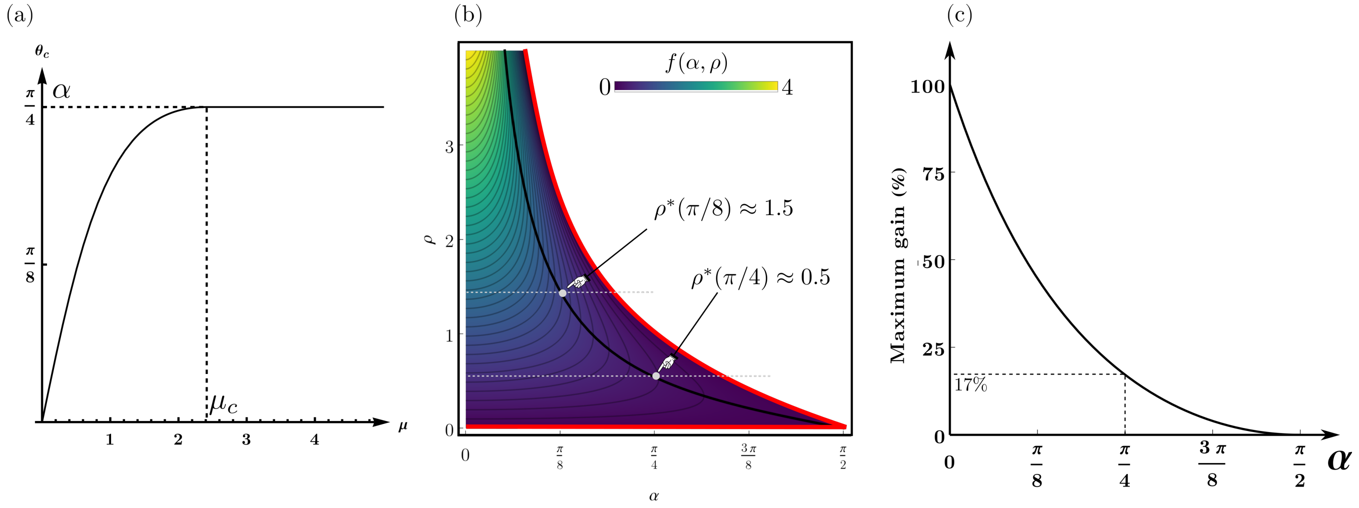


**Fig. S7.** (a) Plot of $\theta_c$ vs $\mu$, for $\alpha = \pi/4$ and for an optimal prey size, i.e. with $\rho = \rho^*(\pi/4)$ [Eq. (26)]. For $\mu > \mu_c$ [Eq. (24)], capture occurs via tumbling. (b) The efficiency function $f(\alpha, \rho)$. Maximal gain corresponds to minimal stability zone $\theta_c$, relative to the point-mass case $\theta_c \approx \mu \ll 1$. (c) For $\mu \ll 1$, the relative gain function $\mathcal{G}(\alpha)$ is independent of $\mu$ and provides the maximum relative decrease of $\theta_c$ with respect to the worst case scenario $\rho \to 0$.

[Fig. S7(a)]. We first remark that $\theta_c$ is independent of the radius of gyration $\gamma$. Secondly, as expected, in the limit $\rho \to 0$ (with $\theta \leq \alpha$), we also retrieve the classic result of point-mass Coulombian friction, namely $\tan\theta_c = \mu$ (2). The latter happens to correspond to the worst-case scenario (from the plant's point of view), i.e. maximal $\theta_c$. We obtain the same $\theta_c$ for $\rho \to \cot\alpha$ (Eq. (19)) where the leg axis is tangent to the surface. The second case [Eq. (25)] corresponds to the situation where the reaction force at one contact point becomes negative, i.e. contact is lost and the prey tumbles into the traps (in reality, arthropod pads have some degree of adhesion, but we ignore this aspect for simplicity, arguing that adhesion is largely suppressed by the wetting of the peristome). For large friction coefficients $\mu > \mu_c$ (given $\rho$ and $\alpha$), slipping becomes impossible and capture may only occur via tumbling. In summary, a prey $(\alpha, \rho)$ satisfying the non-penetration constraint Eq. (19) will be in equilibrium if $\theta \leq \theta_c$, with $\theta_c$ described by Eqs. (23) to (25).

**C. Optimal prey size.** To explore the functional role of peristome size, we fix the size of the peristome and we look for the typical size of the most unstable prey; in other words, we consider the value of $\rho$ that

minimizes $\theta_c$ (for any fixed angle $\alpha$). Remarkably, we show that this minimum is attained at a *finite* value of $\rho$, given by

$$\rho^*(\alpha) = \sqrt{\frac{\csc\alpha(\csc\alpha - 1)}{2}}, \qquad [26]$$

which, interestingly, is independent of $\mu$ (only the value of the minimum will depend on $\mu$). For a slippery peristome ($\mu \ll 1$), the leading order expansion of Eq. (23), namely

$$\theta_c = \frac{\mu}{1 - \rho^2\sin^2\alpha + \rho\cos\alpha\sqrt{1-\rho^2\sin^2\alpha}} + \mathcal{O}\left(\mu^3\right), \qquad [27]$$

further shows that, to second order in $\mu$, the relative gain in capture efficiency $\theta_c/\mu$, with respect to the worst case $\rho \to 0$, is only dependent on the geometry, via the fundamental efficiency function

$$f(\rho,\alpha) = \rho\cos\alpha\sqrt{1-\rho^2\sin^2\alpha} - \rho^2\sin^2\alpha \qquad [28]$$

[Fig. S7(b)]. Maximum $f$ corresponds to maximum efficiency gain, given by $\mathcal{G}(\alpha) = 1 - \theta_c(\alpha, \rho^*(\alpha))/\theta_{c,\max} = (1-\sin\alpha)/(1+\sin\alpha)$ [Fig. S7(c)]. The black solid line in Fig. S7(b) shows the path of maximum efficiency (for all fixed values of $\alpha$) along the surface $f(\alpha,\rho)$. For example, for a realistic angle $\alpha = \pi/4$ [red dashed line in Fig. S7(b)], the efficiency is maximal for $\rho^*(\pi/4) = \sqrt{1 - 1/\sqrt{2}} \approx 0.54$, which generates a relative efficiency gain $1 - \theta_c^*/\mu = 3 - 2\sqrt{2} \approx 17\%$ with respect to the point mass scenario, irrespective of the value of $\mu \ll 1$. Conversely, for any insect size $\rho$, the maximal capture efficiency is attained for $\alpha \to 0$. In this case, however, the insect will tumble and fall inside the pitcher before slipping.

## References


1. Wolfram Research, Inc. (2022) Mathematica, Version 13.2.
2. Johnson KL (1987) *Contact mechanics.* (Cambridge university press).
3. Erdmann M (1993) Multiple-point contact with friction: Computing forces and motions in configuration space in *1993 International Conference on Intelligent Robots and Systems.* (IEEE), pp. 163–170.
4. Erdmann M (1994) On a representation of friction in configuration space. *The International Journal of Robotics Research* 13(3):240–271.
